# Optical convolutional spectrometer

**DOI:** 10.1038/s41566-026-01891-6

**Published:** 2026-04-15

**Authors:** Chunhui Yao, Jie Ma, Ningning Wang, Peng Bao, Wei Zhuo, Tao Zhang, Wanlu Zhang, Kangning Xu, Ting Yan, Liang Ming, Yuxiao Ye, Tawfique Hasan, Ian White, Richard Penty, Qixiang Cheng

**Affiliations:** 1 Department of Engineering, University of Cambridge, Cambridge, UK; 2 GlitterinTech Limited, Xuzhou, China; 3 University of Bath, Bath, UK

**Keywords:** Integrated optics, Optical sensors, Near-infrared spectroscopy, Optical metrology, Silicon photonics

## Abstract

Optical spectrometers are fundamental across numerous disciplines. However, miniaturized versions, while essential for in situ measurements, are often restricted to coarse identification of signature peaks and are inadequate for metrological purposes. Here we introduce a new class of spectrometer, which uses the convolution theorem as its unique mathematical foundation. Our ‘convolutional spectrometer’ offers unmatched performance for miniaturized systems and distinct structural and computational simplicity, featuring a centimetre-scale footprint for the fully packaged unit, low cost (~US$10) and a 2,400 cm^−1^ (approximately 500 nm) bandwidth in the near-infrared region. We achieve excellent precision in resolving complex spectra with subsecond sampling and processing time, demonstrating wide near-infrared spectroscopic applications from industrial and agricultural analysis to healthcare monitoring. Specifically, our spectrometer system classifies diverse solid samples, including plastics, pharmaceuticals, coffee, flour and tea, with 100% success rate, and quantifies concentrations of aqueous and organic solutions with detection accuracy surpassing commercial benchtop spectrometers. We also realize the non-invasive sensing of human biomarkers, such as skin moisture (mean absolute error = 2.49%), blood alcohol (1.70 mg dl^−1^), blood lactate (0.81 mmol l^−1^) and blood glucose (0.36 mmol l^−1^), highlighting the potential of this new class of spectrometers for low-cost, high-precision, portable and/or wearable spectral metrology.

## Abstract (web summary)

A centimetre-scale convolutional spectrometer offers high performance, low cost and ~500-nm bandwidth. The system classifies diverse solid samples and quantifies concentrations of solutions with detection accuracy surpassing commercial benchtop spectrometers.

## Main

The miniaturization of spectrometers has long been an important pursuit^1–3^. However, current miniaturized spectrometers still face notable performance limitations that hinder their incorporation into embeddable smart devices for real-time, in situ spectroscopic sensing^4,5^. For example, infrared spectroscopy typically necessitates fine resolution, high precision and ultra-wide bandwidth to acquire sufficient spectral information^6,7^. Practical use cases also prioritize other criteria, including noise tolerance, sampling speed, processing complexity and temperature resilience^8,9^. These rigorous, multifaceted requirements are particularly pronounced for healthcare applications^10–12^. Many biomarkers of interest, such as blood lactate and glucose, exist at low concentrations and share overlapped absorption bands, making their data modelling and feature extraction a formidable task^13–15^.

**Fig. 1:**
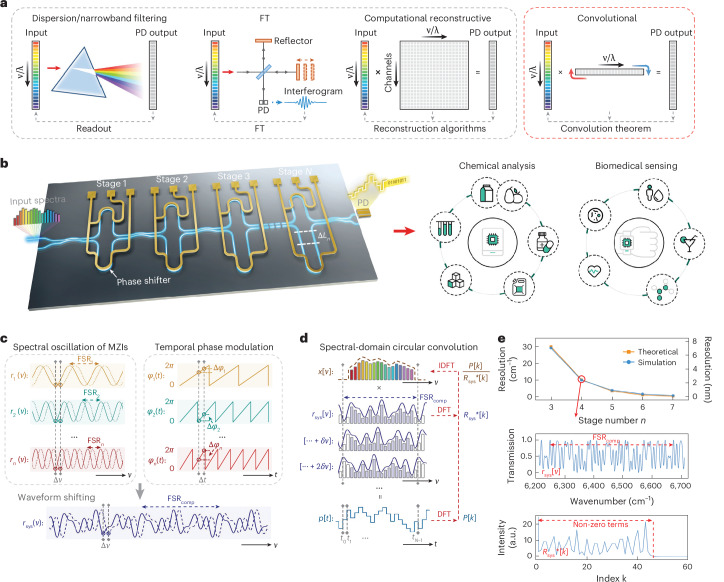
Defining a new class of spectrometer based on the convolution theorem. **a**, Working principles of various spectrometer categories, compared with the proposed convolutional spectrometer. **b**, Schematic (left) of a convolutional spectrometer with cascaded unbalanced MZIs on a photonic integration platform. The conceptual diagram (right) highlights its potential for portable and/or wearable devices, accommodating a wide range of spectroscopic applications. **c**, Diagrammatic illustration of how applying proportional phase modulation to MZI stages with varying FSRs enables spectral-domain waveform shifting of the overlaid system response. **d**, The underlying mechanism of how spectral-domain shifting of the system response executes the circular convolution operation on an incident spectrum, yielding corresponding PD outputs. The right-side arrows illustrate the steps of calculating the convolution theorem for spectrum retrieval. **e**, Spectrometer resolution versus the number of cascading stages (with a fixed composite FSR at 420 cm^−1^). As an example, the bottom two panels show the simulated system response of a four-stage ConvSpec and its corresponding frequency components after DFT. FT, Fourier transform; PD, photodetector; *λ*, wavelength; *r*, spectral response function; *p*, photodetector reading. Source data

Recent advances in photonic integration technologies have paved the way for developing chip-scale spectrometers with mass manufacturability^16,17^. To date, however, reported integrated spectrometers are mostly at bare-die level for proof-of-concept demonstration^18–20^. In general, the underlying operational principles classify existing spectrometers into dispersive, narrowband filtering, Fourier transform and computational reconstruction types (Fig. 1a)^5^. For the first three types, the bandwidth and resolution are inevitably limited by the achievable optical path lengths or path differences, or the scale of the filter arrays, especially for those implemented in planar photonic circuits^21–23^. The reconstructive type, conversely, relies on compressive sensing-based approaches, generally via stochastic sampling and solving underdetermined inverse equations to acquire excessive spectral information^24–26^. Yet, this comes at the cost of notable computational load for reconstruction, non-trivial calibration processes and compromised precision in resolving continuous, non-sparse spectra^27–29^. In addition, the nonlinearity of reconstructive systems makes them highly sensitive to measurement noise, leading to unpredictable spectral distortions^30–32^.

Here we derive from the underlying mathematical foundation and propose a new class of spectrometer, which harnesses the convolution theorem, one of the most fundamental principles in signal processing, to retrieve arbitrary incident spectra. Structurally, the proposed convolutional spectrometer (ConvSpec) requires only a simple cascade of optical components with periodic spectral responses, for example, unbalanced Mach–Zehnder interferometers (MZIs) or micro-ring resonators, to form an overlaid response with systematic periodicity. By applying proportional phase tuning to individual components, the overlaid system response can be linearly shifted in the spectral domain. This waveform shifting physically executes the circular convolution operation on the input spectrum, enabling its recovery via (inverse) Fourier transforms. Such a linear convolutional scheme, owing to its mathematical nature, offers negligible computational load, a relaxed calibration process and strong noise tolerance. Moreover, the system’s inherent periodicity permits the circular convolution to occur within any of its cycles, theoretically granting an unlimited bandwidth. Its resolution can also be exponentially escalated by increasing the number of cascading components and tailoring their individual responses. These features fundamentally distinguish our ConvSpec from all existing types (Table 1).

**Table 1:** Performance characteristics of different spectrometer categories

Principle	Bandwidth	Resolution	Computational complexity	System noise	Detection channel	Fellgett’s advantage
Dispersive/narrowband filtering (spatial)	D.D.	Filter’s FWHM	$O(N)$	$\sigma$	*N*	No
Narrowband filtering (temporal)	D.D.	Filter’s FWHM	$O(N)$	$\sigma$	1	No
Fourier transform (spatial)	$\frac{1}{2\delta {\rm{OPD}}}$	$\frac{1}{{{\rm{OPD}}}_{max}}$	$O(N\log (N))$	$\sqrt{N}\sigma$	$\frac{{{\rm{OPD}}}_{max}}{\delta {\rm{OPD}}}$	No
Fourier transform (temporal)	$\frac{1}{2\delta {\rm{OPD}}}$	$\frac{1}{{{\rm{OPD}}}_{max}}$	$O(N\log (N))$	$\sqrt{N}\sigma$	1	Yes
Computational reconstruction	D.D.	D.D.	$O({N}^{3})$	Nonlinear distortion	D.D.	D.D.
This work: convolutional spectrometer	Unlimited (FSR_comp_ per operation)	$\frac{{{\rm{FSR}}}_{{\rm{comp}}}}{max\{k|{R}_{{\rm{sys}}}[k] > 0\}}$	$O(N\log (N))$	$\sigma$	1	Yes

D.D., design dependent; $\sigma$, standard deviation of the measurement noise; *N*, number of spectral pixels; OPD, optical path difference; FSR_comp_ and *R*_sys_[*k*] denote the composite FSR and the frequency component sequence of system response, respectively (see equations (1)–(6)).

Experimentally, we implement a ConvSpec on a SiN platform and encapsulate it within a comprehensive optoelectronic package that fully integrates the microcontroller unit (MCU), photodiode (PD) and auxiliary electrical circuits. Our device achieves a centimetre-scale overall footprint, a sampling and local processing time of <0.4 s and a total cost of around US$10. Meanwhile, it operates over an ultra-wide near-infrared (NIR) range from 5,900 cm^−1^ to 8,300 cm^−1^ (that is, about 1,200 nm to 1,700 nm), with exceptional accuracy even when resolving sophisticated input spectra. It also exhibits superior thermal robustness thanks to the waveform-shifting mechanism, validated by withstanding an extreme temperature variation from −20 °C to 80 °C.

Our ConvSpec enables real-time, high-precision and data-intensive metrological measurements across various NIR sensing applications. We first showcase the classification of solid substances, such as different types, origins or grades of plastics, pharmaceuticals, coffee, flour and tea, all attaining 100% success. It also measures the concentration and purity of diverse aqueous and organic solutions with ultra-high quantitative precision of around 0.01%, outperforming commercial benchtop spectrometers. Furthermore, our device demonstrates non-invasive sensing of various human biomarkers under dynamic, intricate physiological conditions. It not only realizes the quantitative assessment of skin moisture but also accurately measures low-concentration subsurface biomolecules, including blood alcohol, lactate and glucose. Additionally, it achieves the single-participant long-term tracking of daily glucose fluctuations. This marks the transformation of miniaturized spectrometers into embeddable spectrometric sensors, empowering broad applications from material analysis to healthcare solutions.

## Results

### Principles and theoretical analysis

Figure 1a outlines the underlying principles of current spectrometer types, including spectral demultiplexing with linear readout, interferometry with Fourier transform and sampling matrices with algorithmic reconstruction, together with the proposed ConvSpec that leverages the spectral circular convolution. ConvSpec features a streamlined architecture, requiring only a few optical components with periodic responses. Therefore, it can be readily implemented on different material platforms using different building blocks (Supplementary Section 1). Here we employ unbalanced MZIs on a SiN platform as an example (Fig. 1b). Supplementary Fig. 1 further showcases an MRR-based ConvSpec to illustrate the design generality.

Denoting the time-varying spectral response of an individual MZI stage *i* as ${r}_{i}\left(\nu ,t\right)$, where $\nu$ represents the wavenumber and *t* represents time, the cascading system exhibits an overlaid system response ${r}_{{\rm{sys}}}(\nu ,t)$ that equals the product of all individual stage responses. The free spectral range (FSR) of each MZI is given by ${\mathrm{FSR}}_{i}=1/{n}_{{\rm{g}}}{\Delta L}_{i}$, where ${n}_{{\rm{g}}}$ denotes the group index and ${\Delta L}_{i}$ is the arm length difference. Consequently, the cascading system also features a composite FSR, as:

1 $${{\rm{FSR}}}_{\mathrm{comp}}=\mathrm{LCM}[{{\rm{FSR}}}_{i},i=1,2,\ldots {n}_{\mathrm{stage}}]$$

where LCM […] represents the least common multiple. Figure 1c illustrates that by applying phase tuning ${\varphi }_{i}(t)$ inversely proportional to each MZI’s FSR, the overlaid waveform can be circularly shifted over the composite FSR (Methods).

Figure 1d further illustrates how such waveform shifting is utilized to execute the circular convolution. For an incident spectrum $x(\nu )$ that falls within the span of any composite FSR, the temporal output power $p(t)$ is given by:

2 $$p(t)={\int }_{{\nu }_{\mathrm{start}}}^{{\nu }_{\mathrm{end}}}x(\nu ){r}_{\mathrm{sys}}\left(\nu ,t\right)d\upsilon$$

where ${{\nu }_{{\rm{end}}}-\nu }_{{\rm{start}}}={{\rm{FSR}}}_{{\rm{comp}}}$. Equation (2) can be discretized into a sequence format, as:

3 $$\begin{array}{rcl}p\left[t\right] & = & \mathop{\sum }\limits_{\nu ={\nu }_{0}}^{{\nu }_{N-1}}x\left[\nu \right]{r}_{\mathrm{sys}}\left[{\left(\nu +\delta v\bullet t\right)}_{N}\right],t=0,1,2,\ldots ,N-1\\ & = & \mathop{\sum }\limits_{\nu ={\nu }_{0}}^{{\nu }_{N-1}}x\left[\nu \right]{{r}_{\mathrm{sys}}}^{* }\left[{(-\nu +\delta v\bullet t)}_{N}\right],t=0,1,2,\ldots ,N-1\end{array}$$

where $\delta \nu$ represents the smallest footstep in waveform shifting, $t$ is discretized time index and ${(\bullet )}_{N}$ denotes the circular shift of the sequence modulo *N* (${N={\rm{FSR}}}_{{\rm{comp}}}/\delta v$). ${{r}_{\mathrm{sys}}}^{* }[\nu ]$ refers to the flip of the original sequence ${r}_{\mathrm{sys}}[\nu ]$. Notably, equation (3) strictly adheres to the circular convolution theorem^33^, allowing its transformation via discrete Fourier transform (DFT) to yield:

4 $$P[k]={{R}_{{\rm{sys}}}}^{\ast }[k]X[k],k=0,1,\ldots ,N-1$$

where $k$ denotes the sequence index. Therefore, the incident spectrum can be retrieved through the inverse DFT (IDFT), as:

5 $$x[\nu ]=\mathrm{IDFT}\left(\frac{P[k]}{{{R}_{\mathrm{sys}}}^{\ast }[k]}\right)=\mathrm{IDFT}\left(\frac{\mathrm{DFT}({\rm{p}}[{\rm{t}}])}{\mathrm{DFT}({{{\rm{r}}}_{\mathrm{sys}}}^{\ast }[{\rm{\nu }}])}\right)$$

Equation (5) reveals ConvSpec’s key mathematical mechanism, from which two features can be noted: (1) its periodic nature allows the convolution to occur within any composite FSR, whereas the span of composite FSR can be manipulated by tailoring the FSRs of individual MZIs; and (2) as ${{R}_{\mathrm{sys}}}^{* }\left[k\right]$ appears in the denominator, its highest frequency component—that is, the largest non-zero term in ${{R}_{\mathrm{sys}}}^{* }\left[k\right]$—determines the maximum resolvable frequency component, thus defining the spectrometer resolution, as:

6 $${\rm{resolution}}={{\rm{FSR}}}_{{\rm{comp}}}/max\{k|{{R}_{{\rm{sys}}}}^{\ast }[k] > 0\}$$

Hence, it is crucial for the ConvSpec to accommodate a wide, continuous range of frequency components from low to high frequency regions. This can be achieved by systematically optimizing each MZI’s spectral properties and increasing the stage number. Supplementary Section 2 illustrates that increasing stage number leads to exponential growth in system frequency components, enabling an exponential enhancement in resolution as per equation (6). For example, Fig. 1e shows that with the composite FSR set at around 420 cm^−1^ (that is, 100 nm), scaling the number of stages from three to seven enhances the resolution from about 30 cm^−1^ (7 nm) to below 0.5 cm^−1^ (0.1 nm).

Besides, the inherent linearity of convolution ensures that any measurement noise is linearly imposed onto the resolved spectra without amplification or distortion. This allows standard denoising techniques, such as digital low-pass filtering, to effectively improve spectrometer accuracy^34^. In practice, the dispersion effects that stretch the FSR periods can also be compensated by mathematically amending equation (5). Detailed noise and dispersion correction schemes are provided in Supplementary Section 3.

### Device design and characterization

**Fig. 2:**
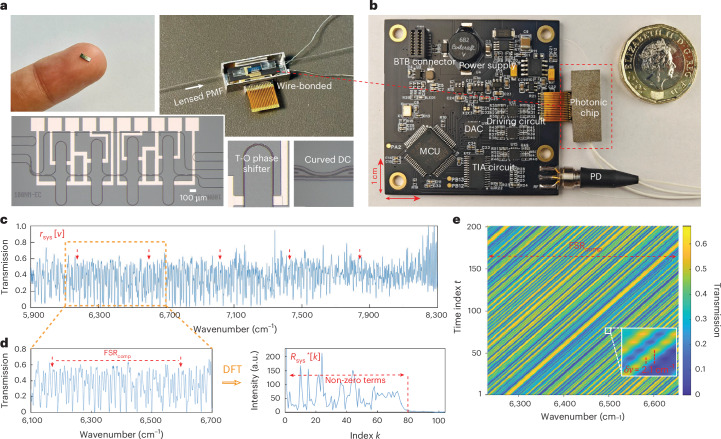
Chip fabrication, packaging and characterization. **a**, Photos of the spectrometer chip, wire-bonded and optically coupled with lensed polarization maintaining fibre. Insets enlarge the thermo-optic phase shifter and curved directional coupler, respectively. **b**, Photo of the fully packaged ConvSpec with a customized printed circuit board. **c**, Measured system response across an ultra-wide spectral range, exhibiting a complex yet periodic waveform. For example, the dashed arrows highlight a series of spectral peaks that repeat over each overlaid FSR of around 420 cm^−1^. **d**, System response within a specific composite FSR and its corresponding frequency components after DFT. The arrows mark the boundaries of this composite FSR. **e**, Measured temporal circular waveform shifting. The inset shows a shifting step of 2.1 cm^−1^. DAC, digital-to-analog converter; TIA, transimpedance amplifier. Source data

Figure 2a,b present images of our ConvSpec chip, mounted on a customized printed circuit board (PCB; Methods). The PCB integrates an MCU for circuit control and local data processing, a fibre-coupled InGaAs PD with readout circuits and auxiliary driving circuits for phase modulation. The packaged device features a compact overall size of 4.8 × 6.2 × 0.6 cm^3^, a light weight of 16.5 g and US$10-level total cost (see breakdowns in Supplementary Table 1). The photonic chip cascades four stages of unbalanced MZIs, offering a reasonable trade-off between the resolution, footprint and control complexity (Supplementary Section 2). Its integration on a SiN platform ensures minimal loss, low dispersion and temperature insensitivity. Curved directional couplers are employed to facilitate an ultra-wide bandwidth (Supplementary Section 5). Figure 2c shows the calibrated system response over a 2,400 cm^−1^ spectral range from 5,900 cm^−1^ to 8,300 cm^−1^ (1,200–1,700 nm), exhibiting a complicated waveform yet with consistent periodicity of around 420 cm^−1^. Such calibration is accomplished by using six superluminescent diodes (SLDs) centred at different wavenumbers as light sources to cover the full bandwidth (Methods). The 2,400 cm^−1^ range is determined by the coverage of the SLDs, whereas the device’s actual working bandwidth is expected to be broader (Supplementary Fig. 6). Figure 2d further plots the system response within a specific composite FSR and its corresponding frequency components, containing abundant non-zero terms up to an index of 78. We calibrate each MZI stage (Supplementary Fig. 7) and apply proportional phase modulations (Methods). Figure 2e presents the measured circular waveform shifting over time, showing excellent uniformity and smoothness in shifting.

For performance characterization, we conduct the convolution operation across an overlaid FSR between 6,250 cm^−1^ and 6,670 cm^−1^ (1,500–1,600 nm), utilizing a benchtop waveshaper (Coherent WS-1000B) to generate diverse incident spectra. Here the shifting step $\delta v$ is set at 2.1 cm^−1^ (that is, *N* = 200; Fig. 2e), which ensures the Nyquist criterion by keeping the sampling resolution finer than half the system resolution. This imposes a negligible computational load, requiring only fast Fourier transforms on a 200-point sequence, which can be calculated by the on-board MCU in less than 50 ms (<0.1 ms on a typical laptop).

**Fig. 3:**
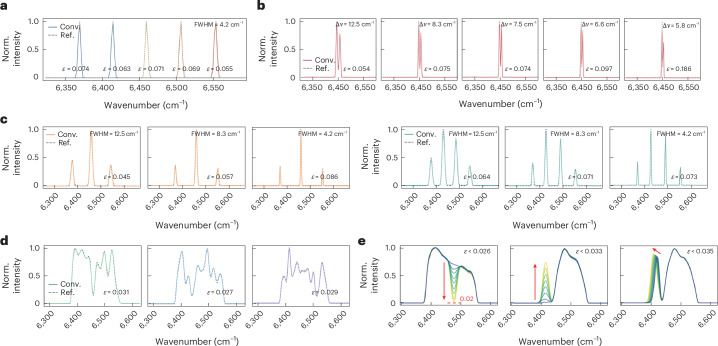
Performance under diverse spectral inputs. **a**, Resolved single-peak spectra centred at varying wavenumbers. **b**, Resolved dual-peak spectra with decreasing spectral spacing. **c**, Resolved tri- and quad-peak spectra with different FWHMs. **d**, A few examples of the resolved continuous, randomly shaped spectra; see more results in Supplementary Fig. 13. **e**, Resolved continuous spectra with gradually dipping, rising and shifting peaks, respectively. Norm., normalized. Source data

We first examine various discrete, narrowband spectra, including single-, dual-, triple- and quad-peak signals with different positions and full-width at half-maximum (FWHM; Fig. 3a–c). The spectral accuracy is quantified by the L2-norm relative error *ε* (Methods). Notably, the spacing of dual-peak signals is reduced down to 5.8 cm^−1^ (1.4 nm), validating such resolution given the Rayleigh criterion. This closely matches the theoretical calculation based on equation (6) (that is, 420 cm^−1^/78 = 5.4 cm^−1^). Subsequently, a range of continuous broadband spectra with randomly shaped waveforms are tested (Fig. 3d and Supplementary Fig. 13). Our device accurately captures those complicated spectral features, including sharp peaks with rapid roll-offs and minor bumps, with ultra-low relative errors between 0.022 and 0.044. Moreover, we create waveform groups with gradually varying peaks. Figure 3e presents the resolved waveforms with dipping, rising and shifting characteristics, respectively, all achieving relative errors lower than 0.035. Notably, peak intensity as low as 0.02 is identified (relative to the maximum of 1.0), demonstrating a dynamic range of over 17 dB.

Thanks to the circular shifting nature, ConvSpec can readily compensate for thermally induced spectral shifts, allowing nearly infinite temperature tolerance. For validation, the device is placed in a temperature chamber with ambient temperature varied from −20 °C to +80 °C. Supplementary Section 7 elaborates that this variation only causes a waveform shift of approximately 0.016 nm °C^−1^, which can be counteracted by applying algorithmic correction, illustrating tolerance of at least 100 °C.

### Spectrometric analysis of solid and liquid substances

**Fig. 4:**
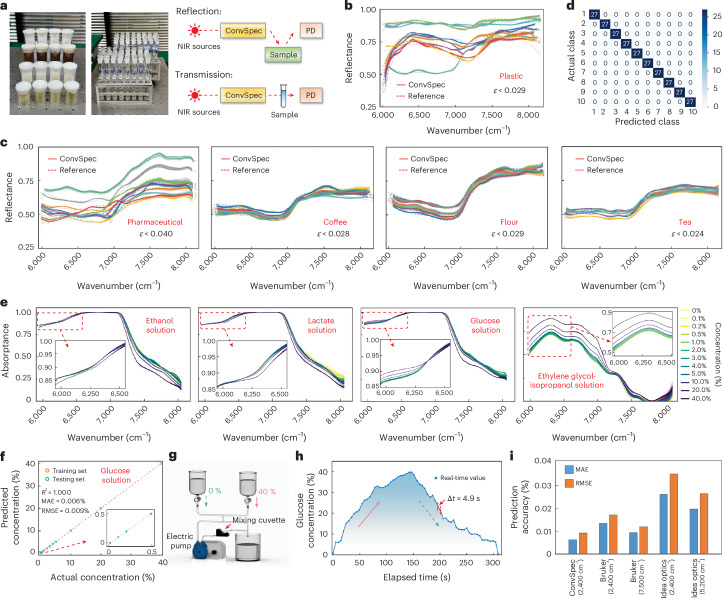
Spectrometric measurement of various solid and liquid samples. **a**, Photos of some representative solid and solution samples under test (left) and diagrams illustrating the experimental setups for solid reflectance and liquid transmittance measurements (right). **b**, Measured reflectance spectra of different plastic samples (80 repetitions per sample), with references measured using a benchtop spectrometer. **c**, Measured reflectance spectra of different pharmaceuticals, coffee, flour and tea, respectively. **d**, Confusion matrix of the classification results, using plastic samples as an example. **e**, Measured absorptance spectra of aqueous solutions (ethanol, lactate, glucose) and organic solutions (ethylene glycol in isopropanol) across concentrations from 0.1% to 40% (80 repetitions per concentration). **f**, The predicted concentrations via the SVR model versus the actual concentrations, using glucose solution measurements as an example. **g**, Schematic of a dynamic solution-mixing system with two solutions of different concentrations being injected. **h**, Real-time tracking of glucose solution concentration using a pretrained SVR model, achieving a system-level time resolution of 4.9 s. Solid and dashed arrows denote the injection of high- and low-concentration solutions, respectively. **i**, Comparison of concentration prediction accuracy between our device and commercial benchtop spectrometers in glucose solution tests, using their partial and full working bandwidths for data acquisition, respectively. Source data

ConvSpec empowers the spectrometric analysis of various substances via reflection and transmission measurements (Fig. 4a). First, we demonstrate the classification of solid samples, ranging from different types of plastics and pharmaceuticals to coffee, flour and tea of varying grades and qualities (Methods). Such identifications are critical for applications across industrial production, recycling and food safety—such as distinguishing reusable plastics^35^ or detecting gluten content in flour products^36^—yet remain challenging via visual inspection. Each sample is measured 80 times, with the data randomly split into training and testing sets in a 2:1 ratio. Here we also employ the six SLDs for sequential illumination (Supplementary Fig. 9). Figure 4b,c plot the reflectance spectra for plastic and all other samples, with relative errors lower than 0.029, 0.040, 0.028, 0.029 and 0.024, respectively. Our device exhibits exceptional stability, with the standard deviation of 80 consecutive measurements ranging between 0.001 and 0.004 across samples. Classification models are trained using the *k*-nearest neighbours (kNN) algorithm, all realizing 100% success (Fig. 4d and Supplementary Fig. 14).

We also showcase the concentration analysis of liquids, including aqueous solutions of ethanol, lactate and glucose, and organic solutions of ethylene glycol in isopropanol, all prepared at gradient concentrations from 0.1% to 40%. These tests validate the device’s ultra-high sensitivity and underscore its applicability in chemical and pharmaceutical practices^37^. Figure 4e displays the absorptance spectra of different solutions, each measured 80 times. Corresponding models are trained with a support vector regression (SVR) algorithm to predict concentrations, all achieving a coefficient of determination (*R*^2^) of 1.000. These models exhibit exceptional accuracy on their testing sets, yielding mean absolute errors (MAEs) of 0.017%, 0.015%, 0.006% and 0.006%, and root mean squared errors (RMSEs) of 0.022%, 0.021%, 0.009% and 0.013%, respectively (Fig. 4f and Supplementary Fig. 15).

To assess system-level response speed, we construct a dynamic solution-mixing setup (Fig. 4g). Figure 4h illustrates that our device, together with the pretrained SVR model, tracks the real-time glucose concentrations at a second-scale interval (Supplementary Video 1). This is primarily attributed to the SVR inference, as the device’s intrinsic sampling and processing time is below 0.4 s (Methods). The glucose concentration tests are also replicated using commercial products: a dispersive spectrometer (IdeaOptics NIR17+Px) and a Fourier-transform spectrometer (Bruker MPA II). Figure 4i illustrates that our ConvSpec achieves even lower MAE and RMSE, owing to its measurement stability and noise tolerance (Supplementary Section 11).

### Non-invasive sensing of human biomarkers

**Fig. 5:**
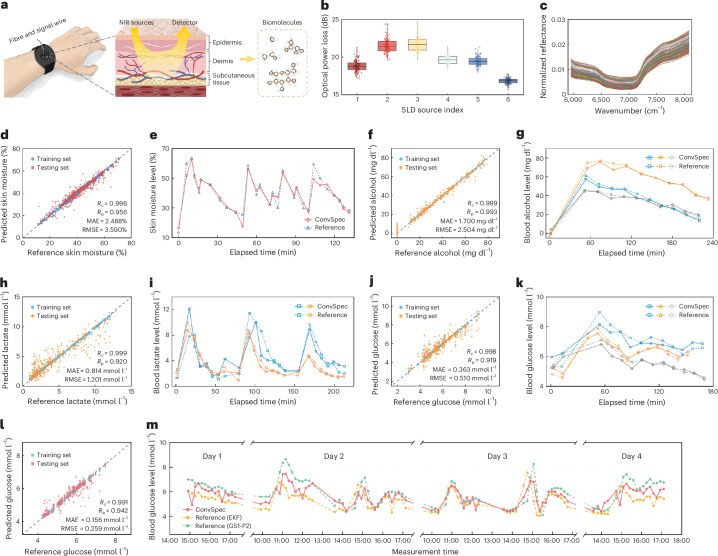
Non-invasive sensing of human biomarkers. **a**, Conceptual diagram of conducting NIR spectroscopic analysis of various biomarkers using our ConvSpec with a wearable probe. **b**, Distributions of optical power losses measured from 126 participants under illumination by 6 different SLD sources. Box plots show the median (centre line) and interquartile range (box), with whiskers extending to the minimum and maximum values. **c**, Preprocessed skin reflectance spectra from different participants at varying skin moisture levels, normalized to the SLD sources. **d**, Predicted versus reference skin moisture values based on an SVR model. **e**, Real-time tracking of a new participant’s skin moisture levels across four consecutive rise-and-fall cycles using the pretrained model. **f**,**h**,**j**, Predicted versus reference concentrations of blood alcohol (**f**), lactate (**h**) and glucose (**j**) based on respective SVR models. **g**,**i**,**k**, Real-time variations in blood alcohol (**g**), lactate (**i**) and glucose (**k**) concentrations from specific participants, compared with reference values obtained from conventional physicochemical approaches. Different symbols (squares, circles and diamonds) denote different participants. **l**, Predicted versus reference concentrations of blood glucose based on a single-participant DFNN model. **m**, Long-term monitoring of daily glucose concentration fluctuations. Source data

Beyond substance analysis, we deploy ConvSpec as a non-invasive sensor in dynamic, individual-diverse physiological contexts. A wrist-wearable probe is developed as the sampling interface (Fig. 5a and Supplementary Fig. 10). This setup allows a millimetre-scale penetration depth, capturing the reflectance spectra from epidermis, dermis and even subcutaneous tissues^38,39^ (Supplementary Section 9). To establish correlations between complex human spectra and faint biomarker signals, independent controlled-variable experiments are conducted (Methods), involving *n* = 126 participant sessions in total and yielding around 6,000 full-band spectra (statistics in Supplementary Section 12). Figure 5b illustrates the optical losses measured across all participants in their initial experimental states under six SLD sources, ranging from 16 dB to 22 dB (that is, ~1–3% of incident power captured; see system noise analysis in Supplementary Section 10). The interindividual variations are attributed to differences in skin tone, body fat percentage and other physiological factors.

We first examine the sensing of skin moisture (Fig. 5c), analysing spectra measured from 26 participants with varying moisture levels (Methods). The corresponding multi-participant SVR model achieves a corrected and prediction correlation coefficient (that is, ${R}_{{\rm{c}}}$ and ${R}_{{\rm{p}}}$) of 0.996 and 0.952, respectively. Figure 5d plots the predicted versus reference values of skin moisture, realizing an MAE of 2.49% and RMSE of 3.59%. Supplementary Fig. 17a shows the distribution of prediction errors with an 80th percentile at 3.98%. To validate the model’s transferability, additional measurements are performed on a new participant. The pretrained model accurately tracks moisture fluctuations between about 10% to 60%, delivering an MAE of 2.61% and RMSE of 3.62% (Fig. 5e).

We then investigate the subsurface detection of critical blood metabolites, which is particularly challenging due to their low concentrations and subtle molecular signatures^40,41^. For blood alcohol, we measure spectra from 24 participants throughout their individual progression from sobriety to mild intoxication (Supplementary Fig. 18b). An SVR model is trained to predict alcohol levels, achieving an MAE of 1.70 mg dl^−1^ and RMSE of 2.50 mg dl^−1^ (Fig. 5f). Figure 5g showcases the real-time alcohol variations from three representative participants, exhibiting peak values between 60–80 mg dl^−1^ that gradually decline to 20–40 mg dl^−1^ over 3 hours. Likewise, blood lactate sensing is assessed by collecting spectra from 48 participants undergoing intense anaerobic exercise (Supplementary Fig. 18c). Figure 5h plots the modelling results, with an MAE and RMSE of 0.814 mmol l^−1^ and 1.201 mmol l^−1^, respectively. Figure 5i presents the temporal variations in lactate concentration from two participants, showing distinct peaks and valleys that correspond to three consecutive exercise sessions. The modelling accuracy for lactate can be further enhanced by incorporating heart rates as an additional data dimension, reducing the MAE and RMSE to 0.454 mmol l^−1^ and 0.669 mmol l^−1^, respectively (Supplementary Section 14).

Importantly, ConvSpec demonstrates its potential in non-invasive blood glucose monitoring—a long-standing challenge in the field^42–44^. Supplementary Fig. 18d shows the spectra from 27 participants undergoing a standard oral glucose tolerance test (Methods)^13^. The resulting SVR model achieves an MAE and RMSE of 0.363 mmol l^−1^ and 0.510 mmol l^−1^, respectively (Fig. 5j). Figure 5k exemplifies three participants’ glucose variations during the oral glucose tolerance test. Moreover, we perform a long-term trial to monitor the daily glucose fluctuations of one particular participant. This task is profoundly difficult, as the subtle dietary glucose variations can be easily overwhelmed by the physiological changes over time. Accordingly, a personalized model is developed using a deep feedforward neural network (DFNN) to enhance feature extraction (Methods), which delivers an MAE and RMSE of 0.156 mmol l^−1^ and 0.259 mmol l^−1^, respectively (Fig. 5l). Figure 5m presents the retrieved glucose fluctuations over multiple days, realizing mean absolute relative differences of 7.82% and 10.03% against two references. Given the current data scale, neither the trained multi-participant nor single-participant glucose model is transferable to new participants, as interindividual variability compromises the model correlation (Supplementary Section 15). This limitation must be addressed by greatly expanding the database.

## Discussion and conclusion

Table 1 summarizes the characteristics of ConvSpec against other spectrometer types, highlighting its comprehensive advantages in terms of bandwidth, precision, computational complexity, noise tolerance, structural simplicity and optical throughput. Supplementary Table 3 benchmarks our device against state-of-the-art miniaturized spectrometers based on various principles and platforms, underscoring its superior performance, technical readiness and cost-effectiveness. Furthermore, Supplementary Table 4 reviews a variety of NIR spectroscopic studies using portable and benchtop spectrometers or prototype-level sensors, illustrating that our spectrometer delivers modelling performance comparable to commercial instruments.

In summary, we define a new class of spectrometer based on the convolution theorem and showcase diverse applications from chemical analysis to non-invasive biomedical sensing. This represents a substantial leap towards low-cost, high-performance and embeddable spectrometric sensors, unlocking new possibilities from industrial scenarios to next-generation healthcare solutions.

## Methods

### Circular waveform shifting in the spectral domain

For an unbalanced MZI *i*, its periodic response is written as^45^:

7 $${r}_{i}\left(\nu ,t\right)={\rho }^{2}+{\left(1-\rho \right)}^{2}+2\rho \left(1-\rho \right)\cos \left(2\pi {n}_{\mathrm{eff}}{\nu \Delta L}_{i}+{\varphi }_{i}(t)\right)$$

where $\rho$ is the power splitting ratio of directional couplers. The oscillation term $\cos \left(2\pi {n}_{\mathrm{eff}}{\nu \Delta L}_{i}+{\varphi }_{i}(t)\right)$ is dictated by both arm length difference ${\Delta L}_{i}$ and temporal phase shift ${\varphi }_{i}(t)$. Accordingly, the overlaid system response can be denoted as:

8 $${r}_{\mathrm{sys}}\left(\nu ,t\right)=\mathop{\prod }\limits_{i=1}^{{n}_{\mathrm{stage}}}({\gamma }_{1}({\gamma }_{2}+\cos (2\pi {n}_{\mathrm{eff}}{\nu \Delta L}_{i}+{\varphi }_{i}(t))))$$

where ${\gamma }_{1}=2\rho \left(1-\rho \right)$ and ${\gamma }_{2}={(\rho }^{2}+{\left(1-\rho \right)}^{2})/2\rho \left(1-\rho \right)$. As shown by Fig. 1c, the time-varying phase shift at each MZI ${\varphi }_{i}(t)$ introduces a circular waveform shift within its FSR. Likewise, when the variations in phase shift across different MZI stages are proportional to their respective arm length differences, that is, adhering to the following equation:

9 $$\begin{array}{l}\mathrm{mod}\left\{{\Delta \varphi }_{1}\left(t\right):{\Delta \varphi }_{2}\left(t\right):\cdots :\Delta {\varphi }_{N}\left(t\right),2\pi \right\}\\ =\frac{1}{{\mathrm{FSR}}_{1}}:\frac{1}{{\mathrm{FSR}}_{2}}:\cdots :\frac{1}{{\mathrm{FSR}}_{i}}=\Delta {L}_{1}:\Delta {L}_{2}:\cdots :\Delta {L}_{i}\end{array}$$

where $\mathrm{mod}\left\{\ldots ,2\pi \right\}$ represents the modulo operation of phase shifts with respect to $2\pi$, the overlaid waveform can be circularly shifted over the composite FSR. This process can be described by reformulating equation (8) in terms of finite differences, as:

10 $$\begin{array}{rcl}{r}_{\mathrm{sys}}\left(\nu ,{t}_{0}+\Delta t\right) & = & \mathop{\prod }\limits_{i=1}^{{n}_{\mathrm{stage}}}\left({\gamma }_{1}\left({\gamma }_{2}+\cos \left(2\pi {n}_{\mathrm{eff}}{\nu \Delta L}_{i}+{\varphi }_{i}\left({t}_{0}+\Delta t\right)\right)\,\right)\right)\\ & = & \mathop{\prod }\limits_{i=1}^{{n}_{\mathrm{stage}}}\left({\gamma }_{1}\left({\gamma }_{2}+\cos \left(2\pi {n}_{\mathrm{eff}}{(\nu +\Delta \nu )\Delta L}_{i}+{\varphi }_{i}\left({t}_{0}\right)\right)\,\right)\right)\\ & = & {r}_{\mathrm{sys}}\left(\nu +\Delta \nu ,{t}_{0}\right)\end{array}$$

where $\Delta \nu =\frac{\Delta {\varphi }_{i}\left(\Delta t\right)}{2\pi {n}_{\mathrm{eff}}{\Delta L}_{i}}$. Equation (10) elucidates how the ConvSpec converts time-domain phase modulation into a spectral-domain waveform shifting.

### Chip fabrication and packaging

The spectrometer chip was fabricated on the CORNERSTONE SiN integration platform, which employs standard deep ultraviolet lithography with a 250-nm feature size. The platform comprises a 300-nm-thick SiN layer sandwiched between a 3-µm buried oxide layer and a 2-µm silicon dioxide top cladding layer. The chip, with a footprint of 0.9 × 3.4 mm^2^, was wire-bonded for electrical fan-out and edge-coupled to lensed polarization maintaining fibres for optical assessment (Fig. 2a). Ultraviolet-curable adhesive was applied to secure the fibres and other components for mechanical robustness, realizing a coupling loss of around 2.5 dB per facet. A thermoelectric cooler was placed underneath the chip, which operated in tandem with a thermistor to realize temperature stabilization.

### Printed circuit board design

A compact PCB was developed to facilitate full packaging of the spectrometer chip, as shown in Fig. 2b. The PCB integrated an MCU (STM32), a fibre-coupled InGaAs PD (LSIPD-L0.3) with transimpedance amplifier readout circuits, driving circuits based on 8-channel digital-to-analog converters and operational amplifiers, as well as the associated power management module and data communication port. The MCU controlled the driving voltages and calculated the convolution theorem for spectra recovery, achieving a total sampling and processing time of less than 0.4 s per spectrum.

### Optical testbed and sampling schemes

To calibrate the system spectral response (Fig. 2c), we sequentially launched six SLDs (Suna Optoelectronics, high-power SLD series) located at different wavenumbers as broadband light sources and measured the corresponding output spectra via a commercial benchtop spectrum analyser. The obtained full-band system response was then stored in the MCU. During the spectroscopic sensing of various substances and human biomarkers, these SLDs were also sequentially turned on for sample illumination. To accommodate their bandwidths, we utilized another chip variant with the overlaid FSR doubled to 840 cm^−1^. This enables the convolutional spectral recovery within each SLD’s coverage range, thereby forming the sample’s full-band reflectance and absorbance spectra. The schematic of the testbed workflow and the amplified spontaneous emission spectra of the SLDs are shown in Supplementary Fig. 9.

For fibre-confined signals (for example, those in Fig. 3), measurements were taken using the fibre-coupled PD on the PCB. The L2-norm relative error *ε* was used to quantify the recovery accuracy, defined as $\varepsilon ={{||}{\varPhi }_{0}-\varPhi {||}}_{2}/{{||}{\varPhi }_{0}{||}}_{2}$ where $\varPhi$ and ${\varPhi }_{0}$ denote the resolved and reference spectra, respectively. Meanwhile, different free-space sampling interfaces were developed to facilitate our spectrometric experiments. Supplementary Fig. 10a,b details the sampling schemes for measuring the reflectance and absorptance of solid and liquid samples, respectively, where a single-mode fibre lens-based collimator (beam diameter of 500 µm) was used to launch light onto the samples, and InGaAs surface PDs (2 mm diameter; Suna Optoelectronics, PD-2000-C3) were adopted to receive the reflected or transmitted signals. The resulting spectra were then normalized with standard references, that is, a fully reflective whiteboard or an empty cuvette. As for the biomarker sensing, a wrist-wearable probe was customized to measure the reflection spectra from human skin. Supplementary Fig. 10c illustrates the schematic of this probe, featuring a centrally positioned fibre collimator surrounded by identical surface PDs (Suna Optoelectronics, PD-2000-C3) to capture the reflected signal from different skin depths.

### Preparation of solid and liquid samples

All solid samples used in our experiments are common industrial, medical and agricultural products, including 10 types of plastics, 10 varieties of pharmaceutical powders, 10 coffees sourced from different origins, 12 flours with differing gluten content and 8 quality tiers of green tea. Specifically, the 10 plastics include polyethylene terephthalate, polyvinyl chloride, polypropylene, polycarbonate, polyvinyl alcohol, acrylonitrile styrene, acrylonitrile butadiene styrene, polytetrafluoroethylene, polybutylene adipate terephthalate and polyhydroxybutyrate. The 10 pharmaceuticals comprise ibuprofen, paracetamol, povidone-iodine, cefaclor, diclofenac sodium, digestive enzymes, roxithromycin, inosine, riboflavin (Vitamin B2) and glimepiride. Coffee samples are from various regions, such as Yunnan, Colombia, Rwanda and East Java, which also differ in tree species and roasting methods. Flour samples span a range of gluten contents, from low-gluten to high-gluten grades, whereas the green tea samples vary in quality, ranging from standard to premium grades. These samples were ground and sieved into uniformly sized particles and then placed into glass containers for reflectance measurements.

The liquid samples were all carefully prepared at varying concentrations, where the glucose solutions were measured by mass percentage, whereas the ethanol, lactate and ethylene glycol solutions were prepared by volume percentage. These solutions were then transferred into cuvettes for absorptance measurements.

### Data collection procedure for biomarker analysis

To establish datasets correlating human spectra with various biomarkers, we utilized our ConvSpec for spectral collection, while simultaneously employing various physicochemical methods to obtain corresponding reference values from participants. Throughout all biomarker experiments, the skin reflectance spectra were repetitively measured at the same area on the wrist using our wearable probe. Participants were required to keep their forearms relaxed, the skin clean and the probe evenly pressed against the skin surface without excessive pressure or loose gaps. Meanwhile, the temperature and humidity of the testing environment were strictly controlled to ensure data stability and consistency.

During the testing of skin moisture, a commercial conductivity-based moisture meter (Real bubee RBX-916) was used to record the real-time moisture percentage for reference. We first employed an air dryer to reduce skin moisture to below 20% as the starting point for data collection. Afterward, a measured amount of moisturizer was applied to participants’ skin and allowed to absorb for 5 min before being wiped off. Skin moisture and reflectance spectra were then measured simultaneously. This measurement process was repeated every 3 min until no further increase in moisture was observed.

For the blood alcohol testing, a commercial breathalyser (Zhaowei Black-03) was utilized as reference. All participants consumed over 200 ml of high-proof baijiu (42% alcohol by volume) in a semi-satiated state, which ensures peak blood alcohol concentrations of at least 40 mg dl^−1^. Breath alcohol tests were conducted at 15-min intervals from before drinking until 3 h afterward, while skin reflectance spectra were simultaneously recorded using our ConvSpec.

As for blood lactate and glucose, reference values were obtained via fingertip blood samples, analysed using a benchtop lactate-glucose analyser (EKF Biosen C-Line Clinic) via an electrochemical approach. Specifically, in blood lactate experiments, participants first engaged in 60 s to 90 s of intense cycling, which typically elevates blood lactate levels to around 10 mmol l^−1^. Immediately after exercise, fingertip blood samples were taken, while the skin reflectance spectra and heart rate data were also recorded. Such data collection was repeated every 8 min until the blood lactate levels dropped to below 2.5 mmol l^−1^ (around 48 min after exercise). Each participant completed two to three consecutive sessions of such exercise–rest alternation. While in blood glucose experiments, participants engaged in a standard oral glucose tolerance test by consuming 75 g of anhydrous glucose in a fasting state. This led to a rise in their blood glucose levels, typically peaking at about 8 mmol l^−1^, followed by a gradual decline. Fingertip blood samples and skin reflectance spectra were collected at 10-min intervals from before the glucose consumption to 3 h afterward.

During the experiment of long-term blood glucose tracking, the particular participant not only performed the fingertip blood glucose measurements but also wore an invasive needle-inserted glucose probe (GS1-P2, Sibionics) as an additional reference for continuous glucose monitoring. To mimic realistic daily glucose dynamics, the participant was instructed to have multiple meals and snacks throughout the day, whereas other periods mostly involved rest or low-intensity activity (corresponding to the fluctuations shown in Fig. 5m). The amount and type of food intake were not restricted. Sampling of the skin reflectance spectra, fingertip blood and glucose probe readings was performed approximately every 10 min throughout the entire multi-day testing period.

### Statistical analysis of participants and ethical approval

To ensure sample diversity and data richness, we recruited male and female Asian adult participants across various age groups for the biomarker sensing experiments. Supplementary Table 2 details the number of participants, gender ratio and age distribution among various biomarker sensing experiments.

All procedures involving human participants were conducted in accordance with the 1964 Helsinki Declaration and its later amendments or comparable ethical standards. The study protocol was reviewed by the Institutional Ethics Committee of Xuzhou Medical University and deemed exempt from formal ethical review, given its non-invasive and minimal-risk nature. Informed written consent was obtained from all participants. No personally identifiable information or sensitive clinical data were collected or disclosed in this study, and all data were analysed in anonymized form. Those reference experiments involving small-volume fingertip blood collection were performed by experienced professionals, ensuring hygiene and the health of participants throughout the collection process.

### Data processing and modelling

For the spectrometric analysis of solid samples, we develop classification models using the kNN algorithm, which determines the majority class among the *k*-nearest neighbours in the feature space. We iteratively adjust the value of *k* (the number of nearest neighbours) to realize maximal classification accuracy on the training set. The refined model is then evaluated on the testing set. As for predicting solution concentrations, we employ the SVR algorithm, a supervised machine learning technique that maps input data into a high-dimensional feature space and constructs an optimal hyperplane to model nonlinear relationships. We fine-tune the SVR model’s regularization and kernel parameters to optimize the coefficient of determination (*R*^2^) and RMSE on the training set, thereby enabling accurate prediction in the testing set.

During the multi-participant modelling of various biomarkers, we first apply a series of preprocessing steps to improve data quality, including outlier removal, baseline drift correction and moving average smoothing, which help mitigate the impact of system noise and motion-induced instability. The processed data then undergoes the sample set partitioning based on a joint X–Y distances algorithm to ensure uniform distribution between the training and testing sets. Corresponding SVR models are trained, with the radial basis function chosen as the kernel for its effectiveness in capturing nonlinear dependencies. Key parameters are fine-tuned using *R*^2^ and RMSE as optimization metrics. For the single-participant modelling of long-term glucose monitoring, we also preprocess the data before inputting it into a DFNN, which consists of multiple layers of feature extraction and regression sections. Specifically, the feature extraction layers process the input through layers with 512, 256 and 128 nodes, each equipped with batch normalization, LeakyReLU activation and Dropout to enhance stability and prevent overfitting. The regression layers further refine the extracted features using layers of 128, 64 and 32 nodes, and ultimately produce a single output for glucose concentration prediction. The model completes over 3,000 training iterations to ensure prediction accuracy.

In addition, to verify the independence of our models, cross-analysis is performed by transferring data from one model into another. For instance, Supplementary Fig. 21 confirms the independence between the models for blood glucose and lactate. Meanwhile, we evaluate the modelling performance across different algorithms, as detailed in Supplementary Section 18.

### Reporting summary

Further information on the research design is available in the Nature Portfolio Reporting Summary linked to this article.

## Online content

Any methods, additional references, Nature Portfolio reporting summaries, source data, extended data, supplementary information, acknowledgements, peer review information; details of author contributions and competing interests; and statements of data and code availability are available at 10.1038/s41566-026-01891-6.

## Supplementary information

Supplementary Information Supplementary Sections 1–18, Figs. 1–22 and Tables 1–4.

Reporting Summary

Supplementary Video 1 Real-time tracking of glucose solution concentration using our ConvSpec in tandem with a pretrained SVR model.

Supplementary Code 1 Custom code used for different spectroscopic sensing applications, including the kNN and SVR models for solid classification and liquid concentration analysis, and the SVR and DFNN models for biomarker analysis.

## Source data

Source Data Fig. 1 Raw source data underlying Fig. 1.

Source Data Fig. 2 Raw source data underlying Fig. 2.

Source Data Fig. 3 Raw source data underlying Fig. 3.

Source Data Fig. 4 Raw source data underlying Fig. 4.

Source Data Fig. 5 Raw source data underlying Fig. 5.

## Data Availability

All data supporting this study are included within the main text and/or Supplementary Information. Source data are available in the University of Cambridge Repository at 10.17863/CAM.126013. Source data are provided with this paper.

## Code availability

Spectral datasets and associated modelling codes are available from the public repository at GitHub (https://github.com/GlitterinTechRnD/ConvSpec), which include the kNN and SVR models for solid and liquid substances, and the SVR and DFNN models for biomarker analysis.
