# Supplementary material for: Optical convolutional spectrometer
Source: Nat Photonics. 2026 Apr 15;20(6):664–72. doi: 10.1038/s41566-026-01891-6 (PMC13241319; doi:10.1038/s41566-026-01891-6)
Supplement: Supplementary file 1 — Supplementary Sections 1–18, Figs. 1–22 and Tables 1–4. [file 41566_2026_1891_MOESM1_ESM.pdf]

nature photonics

Supplementary information

https://doi.org/10.1038/s41566-026-01891-6

Optical convolutional spectrometer

In the format provided by the authors and unedited

Table of Contents

Section 1 Implementation approaches for convolutional spectrometers ... 2
Section 2 Analysis on bandwidth and resolution ... 3
Section 3 Noise and dispersion correction ... 6
Section 4 Device cost analysis ... 8
Section 5 Design details of curved DC ... 8
Section 6 Thermal phase tuning of MZIs ... 9
Section 7 Temperature stability ... 10
Section 8 Optical testbed and sampling interfaces ... 11
Section 9 Detector placement and sensing depth ... 12
Section 10 System noise analysis ... 13
Section 11 Additional Spectral and modeling results ... 14
Section 12 Statistical summary of participants ... 16
Section 13 Additional data on biomarker sensing experiments ... 17
Section 14 Incorporating heart rate in blood lactate modeling ... 17
Section 15 Additional discussions on the blood glucose model ... 18
Section 16 Review of miniaturized spectrometers and NIR spectroscopy studies ... 19
Section 17 Cross-validation between models ... 23
Section 18 Modeling performance across different algorithms ... 23
Reference ... 24

Section 1 Implementation approaches for convolutional spectrometers

The structural simplicity of the proposed convolutional spectrometer—requiring only a cascade of optical components with periodic responses—allows its flexible implementation across various optical platforms using different building blocks. For instance, in conventional free-space optics, lens systems can be used to cascade multiple stages of interferometers, such as Michelson or Fabry-Pérot interferometers, to construct a convolutional spectrometer. The inherent periodicity of each interferometer ensures a periodic overlaid system response, while the waveform shifting can be readily achieved by adjusting the lens positions. Compared to integrated platforms, this free-space configuration offers advantages such as a larger aperture for light collection, simplified optical coupling, greater flexibility in optical path design, and negligible dispersion effects. Furthermore, miniaturization can be achieved using mature microelectro-mechanical systems (MEMS) technologies[1,2]. Similarly, on optical fiber platforms, one can employ fiber splitters and reflectors to build Michelson interferometers or use fiber Bragg gratings—particularly long-period gratings (LPGs), which provide a wide working bandwidth—to create tunable Fabry-Pérot cavities, thereby realizing the proposed convolutional spectrometers[3–5]. These approaches are both straightforward and cost-effective.

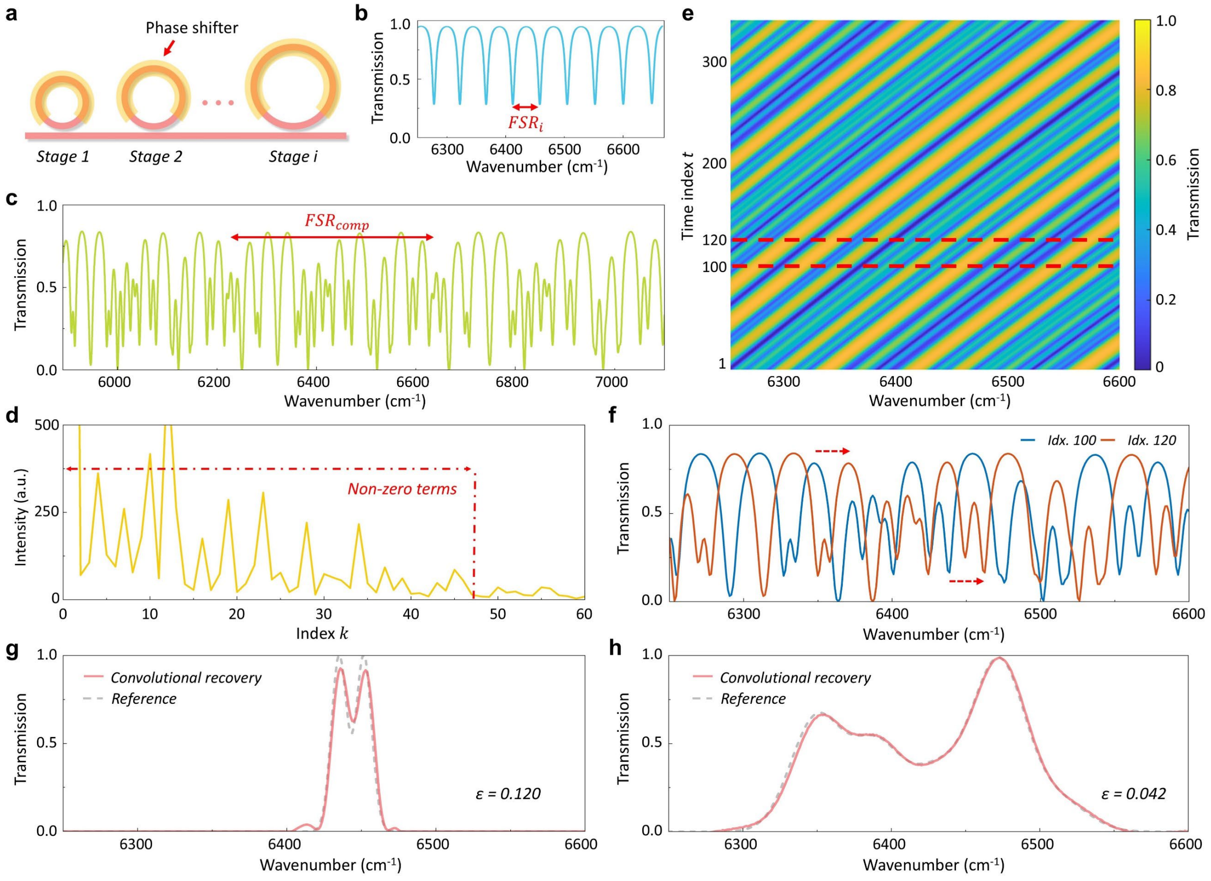

Fig. S1: (a) Schematic diagram of a 4-stage convolutional spectrometer with cascaded all-pass MRRs. (b) Simulated spectral response of an over-coupled MRR. (c) Simulated overlaid system response. The inset shows a composite FSR. (d) Calculated frequency components in the Fourier domain. (e) Simulated temporal circular waveform shifting. (f) System responses at two different time indexes, demonstrating the temporal waveform shifting. (g-h) Resolved dual-peak signal and continuous broadband spectrum, respectively.

For photonic integration platforms, aside from the unbalanced MZI scheme demonstrated in the main text, convolutional spectrometers can also be realized using micro-ring resonators (MRRs) or waveguide-based gratings. Here, we showcase a 4-stage convolutional spectrometer based on all-pass MRRs on a SiN platform, validated through rigorous simulations using Lumerical FDTD and Interconnect. Figure S1a shows the device schematic. Each micro-ring is designed to be over-coupled to attain a wide full-width at half maximum (FWHM) and a moderate extinction ratio, thereby ensuring the system response to feature abundant frequency components with minimal loss[6]. For example, Fig. S1b presents the spectral response of one specific MRR. Figure S1c further depicts the overlaid system response. The corresponding frequency components (Fig. S1d) exhibit around 48 non-zero terms. By applying proportional phase modulation to each MRR following Eq. (9), circular waveform shifting is achieved, as shown in Fig. S1e. Additionally, Fig. S1f presents the system responses at two distinct time points, highlighting the temporal waveform shifting. The spectrometer performance is assessed by resolving various input spectra. For instance, Figs. S1g and S1h illustrate the accurate recovery of a dual-peak spectrum and a continuous, broadband spectrum, respectively.

Besides SiN platform, our designs can be readily transferred to other photonic integration platforms to exploit their respective advantages: for example, silicon-on-insulator (SOI) platforms allow a more compact footprint with enhanced thermo-optic efficiency[7]; Lithium niobate ($LiNbO_3$) platforms offer high-speed modulation owning to its strong electro-optic effect[8]; and III-V monolithic or heterogeneous integration platforms facilitate the incorporation of high-performance PDs and even on-chip light sources, thereby enhancing the system integration level[9,10].

Overall, these results demonstrate the scalability of our convolutional spectrometer under various implementation scenarios.

Section 2 Analysis on bandwidth and resolution

One attractive feature of our convolutional spectrometer is that its periodic nature allows convolution operations to occur within any composite FSR, enabling a theoretically infinite bandwidth. Moreover, as indicated by Eq. (1), the span of composite FSR can be easily customized by tailoring the FSRs of individual MZIs. For example, setting the FSRs of each MZI in a 4-stage system to 420/5 cm^{-1}, 420/7 cm^{-1}, 420/13 cm^{-1}, and 420/17 cm^{-1}, respectively, yields a composite FSR of 420 cm^{-1}. This allows the circular convolution to be performed within any 420 cm^{-1} range, e.g., from 6000 cm^{-1} to 6420 cm^{-1} or from 8000 cm^{-1} to 8420 cm^{-1}. In practice, the composite FSR should be set to exceed the bandwidths of corresponding NIR sources, such as SLDs or LEDs. Therefore, a single convolutional spectrometer can operate in tandem with multiple NIR sources centered at different wavenumbers to cover an ultra-wide detection range.

On the other hand, the resolution of a convolutional spectrometer can be intuitively interpreted through its mathematical principles. As seen in Eq. (5), since ${R_{sys}}^*[k]$ appears in the denominator, the highest frequency component of the system response, i.e., the largest non-zero term in the ${R_{sys}}^*[k]$ sequence, determines the maximum frequency component of the incident spectrum that can be recovered. This theoretically defines the resolution of a convolutional spectrometer, as written by Eq. (6). Therefore, to facilitate superior spectrometer resolution, it is essential for the convolutional core to accommodate abundant frequency components, continuously spanning from low to high frequency regions. In the ideal case without dispersion, the response of a single MZI stage is a triangular function (see Eq. (7)), thus contributing only one frequency component in the Fourier domain, as determined by its arm length difference ΔL_i. However, in a cascaded MZI system, the frequency components arise from the additive and subtractive combinations of the arm length differences across various MZI stages. For example, in a three-

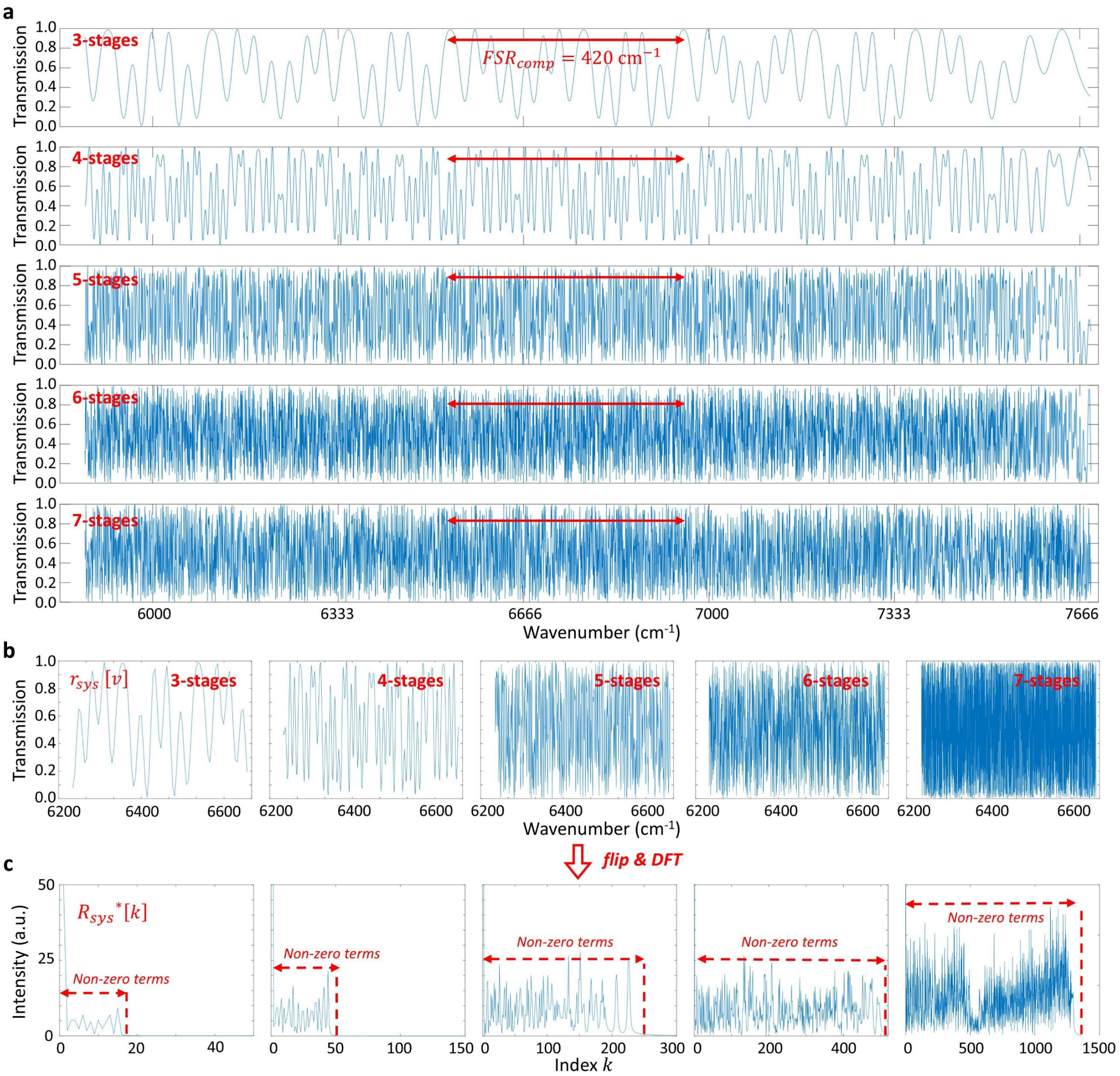

Fig. S2: (a) Simulated system responses for convolutional spectrometers with different numbers of MZI stages, all exhibiting complex overlaid waveforms with consistent periodicity. (b-c) Spectral responses with a specific composite FSR and their corresponding frequency components, respectively, demonstrating the increase in non-zero frequency components with more cascading stages.

stage MZI system, the frequency components are derived from ΔL_1, ΔL_2, ΔL_3, $\Delta L_1 +/- \Delta L_2$, $\Delta L_1 +/- \Delta L_3$, $\Delta L_2 +/- \Delta L_3$, and $\Delta L_1 +/- \Delta L_2 +/- \Delta L_3$. This sums up to 14 components when including the fundamental frequency. In analog, the theoretically maximum number of frequency components $n(k)$ in a cascaded MZI system with N stages can be expressed as:

$$n(k) = 1 + \sum_{i=1}^{N} C_N^i 2^{i-1} \tag{S1}$$

Notably, the term $\sum_{i=1}^{N} C_N^i 2^{i-1}$ is proportional to 3^N, which indicates that as the number of stages increases, the frequency components could grow exponentially, leading to an exponential escalation in resolution.

Hence, to realize optimal system resolution, the arm length difference ΔL_i of various MZI stages should be systematically optimized so that the resulting frequency components in $R_{sys}{}^*[k]$ satisfy two design criteria: 1) avoiding overlap between different spectral components (e.g., to avoid cases such as $\Delta L_1 - \Delta L_2 = \Delta L_3 - \Delta L_2$), and 2) maintaining a continuous coverage with minimal breaks to prevent any loss of spectral information during the convolution process. To achieve this, we employ a traversal algorithm to search for the optimal combination of ΔL_i for convolutional spectrometers with varying stage numbers. Based on these combinations, we simulate their system responses in Lumerical Interconnect. Temporal phase modulations are then applied to simulate the waveform circular shifting. It should be noted that the power splitting ratio ρ in MZIs is also an important design parameter, as it determines the intensities of different frequency components. Our simulations suggest that a ρ between 0.1 and 0.3 facilitates balanced frequency components with suitable intensities. This flexible design space, in turn, provides ample fabrication tolerance for practical implementation.

Figure S2a plots the simulated system responses over an ultra-broad spectral range, showing the complex overlaid waveform with consistent periodicity. Fig. S2b-c further showcases their spectral responses within a specific composite FSR, and corresponding frequency components, respectively. As seen, with the increase in cascading stages, the waveform complexity significantly rises, yielding greater number of frequency components—from less than 20 components in the three-stage system to >1000 components in the seven-stage system. In practice, the fabricated device will exhibit an even richer set of frequency components (see Fig. 2d) due to the dispersion effects, such as variations in the waveguide index and the gradual change in the coupler's power splitting ratio. These effects introduce additional high-frequency components, thereby enhancing the system's resolution.

Based on the simulated spectral responses, we then examine the spectrometer resolution by recovering various dual-peak signals (i.e. following the Rayleigh Criterion). Figure S3 plots the resolved dual-peak

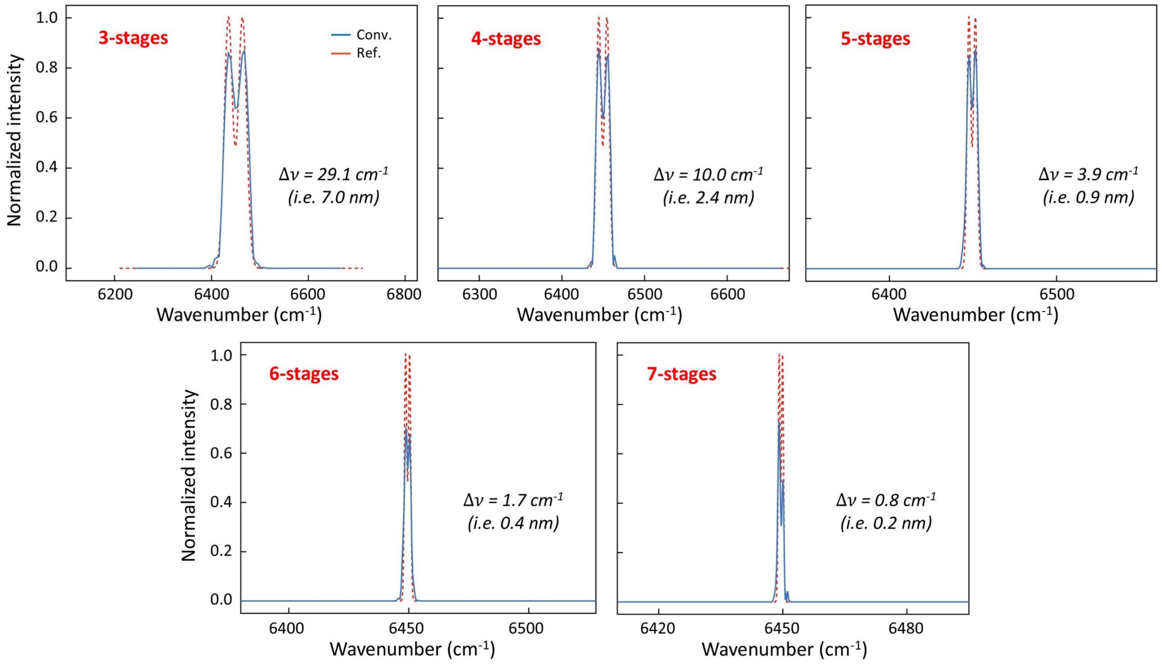

Fig. S3: Simulated recovery of dual-peak signals using convolutional spectrometer with varying numbers of cascading stages.

signals with varying spectral spacings. As the number of stages increases from 3 to 7, the resolution improves from 29.1 cm^{-1} (i.e. 7 nm) to below 0.8 cm^{-1} (i.e. 0.2 nm), which aligns well with the theoretical calculation as per Eq. (6).

In summary, by tailoring the arm length differences and customizing the number of MZI stages, the proposed convolutional spectrometer offers an extensive design flexibility and scalability in terms of bandwidth and resolution, making it adaptable to a wide range of application scenarios with varying performance requirements. Such scaling up, on the other hand, mainly leads to a linear increase in hardware consumption, including chip size, required electrodes, and the number of driving channels, but does not affect the system's control/modulation scheme (see Eq. 9).

Section 3 Noise and dispersion correction

Spectrometers are inherently subject to multiple noise sources, including thermal fluctuations, dark current artifacts, and stray light interference. To quantify these effects, we introduce a temporal Gaussian white noise term $n_{gw}[t]$ with a standard deviation σ into Eq. (3), resulting in:

$$p[t] = \sum_{\nu=\nu_0}^{\nu_{N-1}} x[\nu] r_{sys}{}^*[(-\nu + \delta\nu \cdot t)_N] + n_{gw}[t], \quad t = 0,1,2,\dots,N-1 \tag{S2}$$

Applying the DFT to both sides of Eq. (S2) yields:

$$x[\nu] = IDFT\left(\frac{P[k]-N_{gw}[k]}{R_{sys}{}^*[k]}\right) = IDFT\left(\frac{DFT(p[t])}{DFT(r_{sys}{}^*[\nu])}\right) - IDFT\left(\frac{DFT(n_{gw}[t])}{DFT(r_{sys}{}^*[\nu])}\right) \tag{S3}$$

As observed, due to the presence of noise, the recovered spectrum $x[\nu]$ includes a linear superposition of Gaussian white noise. Since this noise component undergoes both DFT and inverse DFT, its standard deviation remains σ. In other words, the convolutional spectrometer system does not suffer from any spectral distortion, but only linearly adds the measurement noise to the recovered spectrum (i.e. with the noise magnitude remaining unchanged). This linear characteristic allows commonly used noise reduction techniques[11], such as digital low-pass filters, to be utilized to improve recovery accuracy. In contrast, it is worth noting that the reconstructive type of spectrometer exhibits unpredictable nonlinear distortions in the presence of measurement noise. This arises from their reliance on nonlinear reconstruction algorithms, such as iterative convex optimization or neural networks, which essentially perform a global approximation towards the incident spectrum. As a result, noise is not uniformly and linearly superimposed onto the reconstructed spectrum but instead induces random, non-physical artifacts (e.g., spurious side peaks near actual signal features).

Figure S4 showcases the application of a digital low-pass filter during the calculation of Eq. (S3) in a

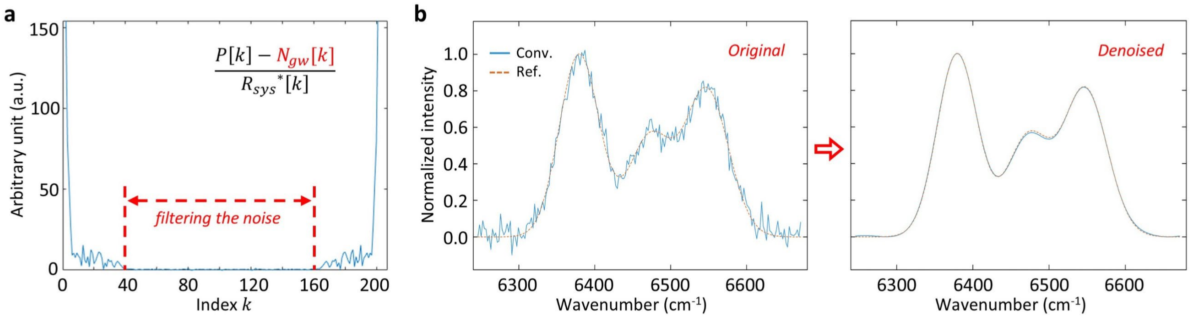

Fig. S4: (a) Calculated signal sequence in the Fourier domain, illustrating the impact of measurement noise. (b) Simulated spectra recovery under a high-noise condition, shown before and after applying a denoising low-pass filter, respectively.

high-noise environment. In Fig. S4a, the calculated term $\frac{P[k]-N_{gw}[k]}{R_{sys}{}^{*}[k]}$ is plotted to reveal the impact of noise in the Fourier domain. Note that here the sequence length N is set to 200, with the highest non-zero frequency component of the system response sequence $R_{sys}{}^{*}[k]$ being 40. This leaves the region from index 40 to 160 containing no spectral information, but only noise. Therefore, a digital filter can be applied to this region to remove the noise. Figure S4b compares the simulated spectral recovery with and without this filtering step, showing a significant noise reduction effect. Advanced denoising algorithms, such as deep neural networks[12], and physical denoising techniques like phase-locked loops[13], can be employed to further mitigate the measurement noise. In practice, prior knowledge of the input light source can also be leveraged to eliminate any spectral artifacts outside its illumination bandwidth.

Besides noise, we investigate the impact of dispersion on our convolutional spectrometer. Specifically, dispersion effects 1) stretch the oscillation period of each MZI stage, and 2) induce variations in phase modulation across the spectral domain. To model such effects, we refer to the previous studies on FT spectrometers, which also fundamentally consist of unbalanced MZIs[14,15]. By denoting the phase shift at central wavenumber ν_0 and time t_0 to be $\varphi_i|_{\nu_0,t_0}$, and assuming that the waveguide's effective refractive index $n_{eff}(\nu)$ follows a series expansion up to first order and the phase modulation varies linearly with wavenumber, the following expressions can be derived[14]:

$$n_{eff}(\nu) = n_{eff}|_{\nu_0} + \partial_\nu n_{eff} u \tag{S4}$$

$$\varphi_i(\nu,t) = \varphi_i|_{\nu_0,t_0} + \Delta\varphi_i(t)(1+\xi)u \tag{S5}$$

where $\partial_\nu n_{eff} = \frac{\partial n_{eff}(\nu)}{\partial \nu}$, $u = \nu - \nu_0$, and ξ is a constant depending on the phase modulation mechanism. For example, in thermal phase shifters, ξ relates to the nonlinearity in thermo-optic coefficient and the thermal expansion of waveguide. Substituting Eq. (S4-S5) into Eq. (10) and expressing it in incremental form yields:

$$\begin{aligned} r_{sys}(\nu,\ t_0+\Delta t) &= \prod_{i=1}^{n_{stage}} \gamma_1\left(\gamma_2 + \cos\left(2\pi n_{eff}(\nu) u \Delta L_i + \varphi_i(\nu,t)\right)\right) \\ &= \prod_{i=1}^{n_{stage}} \gamma_1\left(\gamma_2 + \cos\left(2\pi\left(n_{eff}|_{\nu_0} u + \frac{\partial n_{eff}}{\partial \nu} u^2\right)\Delta L_i\right) + \varphi_i|_{\nu_0,t_0} + \Delta\varphi_i(t)(1+\xi)u\right) \end{aligned} \tag{S6}$$

To eliminate higher-order terms, the mean value theorem can be applied for approximation, i.e., $n_{eff}|_{\nu_0} u + \partial_\nu n_{eff} u^2 \approx (n_{eff}|_{\nu_0} + 2\partial_\nu n_{eff}|_{\nu_0} u_{mid})u$, where $u_{mid} = FSR_{comp}/4$, resulting in:

$$\begin{aligned} r_{sys}(\nu,\ t_0+\Delta t) &= \prod_{i=1}^{n_{stage}} \gamma_1\left(\gamma_2 + \cos\left(2\pi(n_{eff}|_{\nu_0} + 2\partial_\nu n_{eff}|_{\nu_0} u_{mid})(u+\Delta u)\Delta L_i\right) + \varphi_i|_{\nu_0,t_0}\right) \\ &= r_{sys}(\nu+\Delta\nu,\ t_0) \end{aligned} \tag{S7}$$

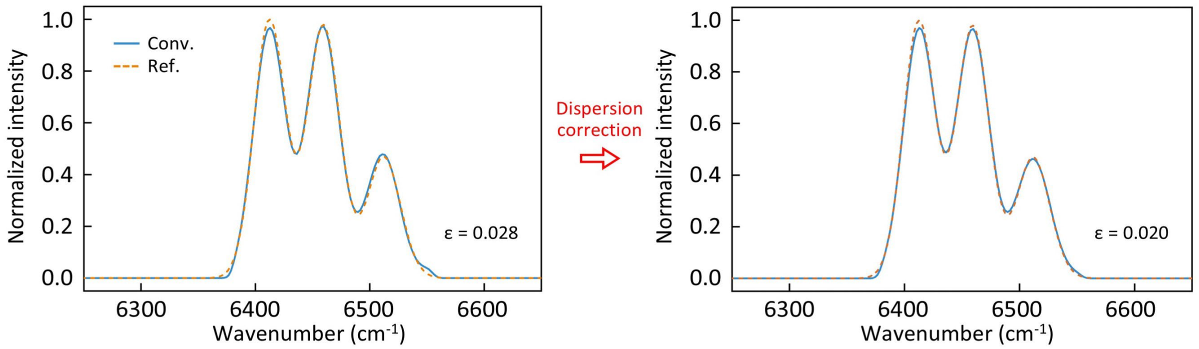

Fig. S5: Resolved spectra with and without the dispersion-correction scheme using experimental data, respectively, showing enhanced spectral accuracy.

where $\Delta u = \Delta \nu = \frac{(1+\xi)\Delta\varphi_i(\Delta t)}{2\pi(n_{eff}|_{\nu_0}+2\partial_\nu n_{eff}|_{\nu_0}u_{mid})\Delta L_i}$. Compared to the ideal non-dispersive case where $\Delta \nu = \frac{\Delta\varphi_i(\Delta t)}{2\pi neff|_{\nu_0}\Delta L_i}$, Eq. (S7) indicates that the presence of dispersion scales the step size in waveform shifting by a factor of $C = \frac{(1+\xi)neff|_{\nu_0}}{n_{eff}|_{\nu_0}+2\partial_\nu n_{eff}|_{\nu_0}u_{mid}}$ when applying the same increment in phase modulation. Thus, during the construction of the discrete convolutional core, this factor needs to be incorporated into the footstep in waveform shifting for dispersion correction, updating Eq. (S7) to:

$$p[t] = \sum_{\nu=\nu_0}^{\nu_{N-1}} x[\nu] r_{sys}^*[(-\nu + \delta\nu^* \cdot t)_N], \quad t = 0,1,2,\ldots,N-1 \qquad \text{(S8)}$$

where $\delta\nu^* = C\delta\nu$. In practice, this correction factor can be easily calibrated by launching a standard incident spectrum as a reference. As an example, Fig. S5 presents the recovery of the same spectrum, with and without the application of the dispersion correction formula, on the basis of experimentally measured data, illustrating the improvement in spectral accuracy.

Section 4 Device cost analysis

Table S1 provides a detailed cost breakdown of our ConvSpec. Currently, leading foundries such as AMF (Singapore), CUMEC (China), and Cornerstone (UK) offer SiN wafer-level tape-out services at approximately \$20,000 per 8-inch wafer (200 mm diameter)[16,17]. Given the compact footprint of our convolutional spectrometer at 0.9 × 3.5 mm^2, a single wafer can produce roughly 10,000 spectrometers, resulting in a cost per photonic chip of less than \$2. This indicates that the integrated photonic chip represents only a small portion of the total sensor cost. In contrast, the control and processing electronics account for a larger portion of the expenses, including an 8-channel DAC (AD5328BRUZ-REEL7) that costs approximately \$3, and an STM32 MCU priced around \$2. The most expensive component is the InGaAs PD (LSIPD-L0.3), which is around \$5 under large-volume procurement. The PCB itself and other components like capacitors and resistors are relatively inexpensive, with a total cost compressed to under \$1.

Note that the above cost analysis is based on current mainstream retail prices, which could decrease further under large-scale production. This highlights the feasibility of mass-manufacturing our design at an ultra-low cost.

Table S1. Device cost breakdown (in USD)

Photonic chip	InGaAs PD	MCU	DAC	PCB and others	Total cost
< 2	< 5	< 2	< 3	< 1	< 13

Section 5 Design details of curved DC

Figure S6a illustrates the schematic of the curved directional coupler (DC) used in constructing the unbalanced MZIs. Compared to conventional symmetrical DC, curved DC offers a better dispersion stability due to its enhanced phase-matching capability[18]. To achieve an ultra-broad operating bandwidth, we systematically optimize its geometrical parameters by runnning a custom particle swarm optimization (PSO) algorithm script in ANSYS Lumerical FDTD[19]. Figure S6b presents the optimized coupling efficiency across wavenumbers, showing that a desired splitting ratio between 0.1 and 0.3 is well maintained across the broad spectral range of 5500 cm^{-1} to 8500 cm^{-1} (i.e. between 1175 nm and 1815 nm), along with a minimal insertion loss. This indicates that the intrinsic bandwidth of our device is expected to be broader

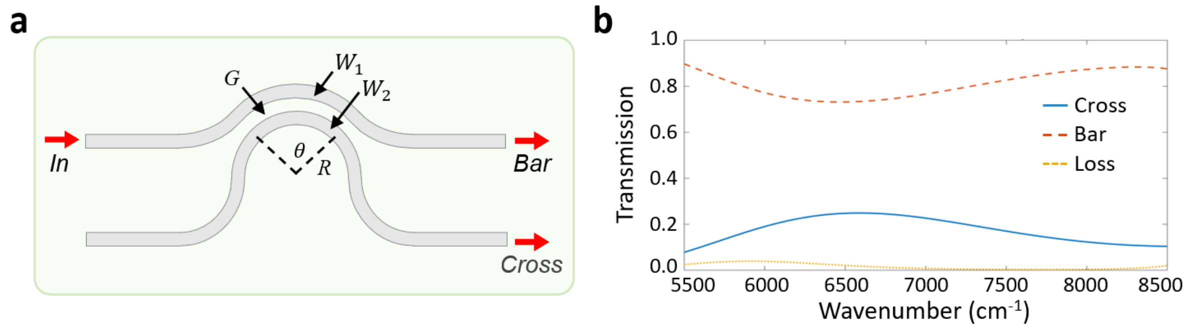

Fig. S6: (a) Schematic of the curved directional coupler. (b) Simulated coupling efficiency over a broad spectral range.

than the currently demonstrated 2400 cm^{-1}. Notably, the minor dispersion in the coupling efficiency does not compromise the convolutional spectrometer's performance. This is because each MZI stage performs spectral shifting within its respective FSR, where the coupling efficiency remains effectively constant over these narrow ranges. Thus, the linear waveform shifting can always be achieved.

Section 6 Thermal phase tuning of MZIs

To enable precise phase modulation, we calibrate all MZI stages by launching a fixed laser signal and sweeping the driving power of each phase shifter while monitoring the output power intensity. For clarity, the driving power is labeled positive for the MZI's long arm and negative for the short arm. Figure S7a illustrates the output power at various driving power levels. Figure S7b further shows the extracted linear relationship between the phase shift and driving power, showing that the complementary phase shifters on both MZI arms achieve the phase modulation from $-\pi$ to $+\pi$ with a thermal tuning efficiency of approximately 32 mW/rad. Accordingly, a look-up table is created to map the targeted phase shift to the corresponding driving power. Note that during our experiments, the smallest phase-shift step is around $\pi/20$, which corresponds to an increment in driving power of about 5 mW. This value is far larger than the

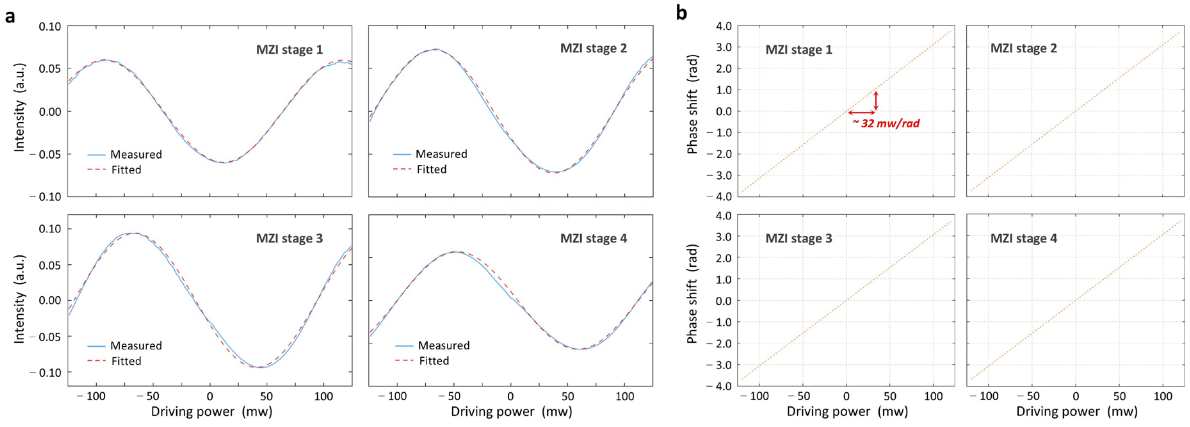

Fig. S7: (a) Measured optical power intensity as a function of the driving power applied to the complementary phase shifter pair on different MZI stages, respectively. (b) Phase shift versus driving power across different MZI stages, showing a consistent thermal tuning efficiency of approximately 32 mW/rad.

power resolution supported by our driving circuits (<0.1 mW), ensuring precise and stable phase modulation during operation. Meanwhile, given that each MZI linearly modulates within the $-\pi$ to $+\pi$ range, the system's average power consumption is around 200 mW.

Section 7 Temperature stability

Temperature variations are typically detrimental to photonic integrated devices, causing red/blue shifts in system responses[20]. However, for the convolutional spectrometer, as the system inherently relies on spectral-domain waveform shifts, it theoretically features an almost infinite temperature tolerance. To validate this, we place our spectrometer inside a temperature chamber and gradually increased the ambient temperature from -20°C to 80°C in 5°C increments. Figure S8a shows the corresponding waveform shift across the ultra-wide spectral range, with insets enlarging a few observation windows. The results indicate that the waveform shift consistently remains at approximately 0.016 nm/°C (equivalent to about 0.055 to 0.095 cm^{-1}/°C across different spectral bands). This moderate spectral shift can be attributed to the low thermal sensitivity of the SiN integration platform.

Accordingly, we model the temperature tolerance by simulating spectral recovery under varying temperatures. Figure S8a displays the output PD signal sequences $p[t]$ for a specific input spectrum across different temperatures. As observed, temperature drift primarily induces a cyclic shift in the intensity sequences, accompanied by minor amplitude variations. Therefore, in practice, a thermistor can be

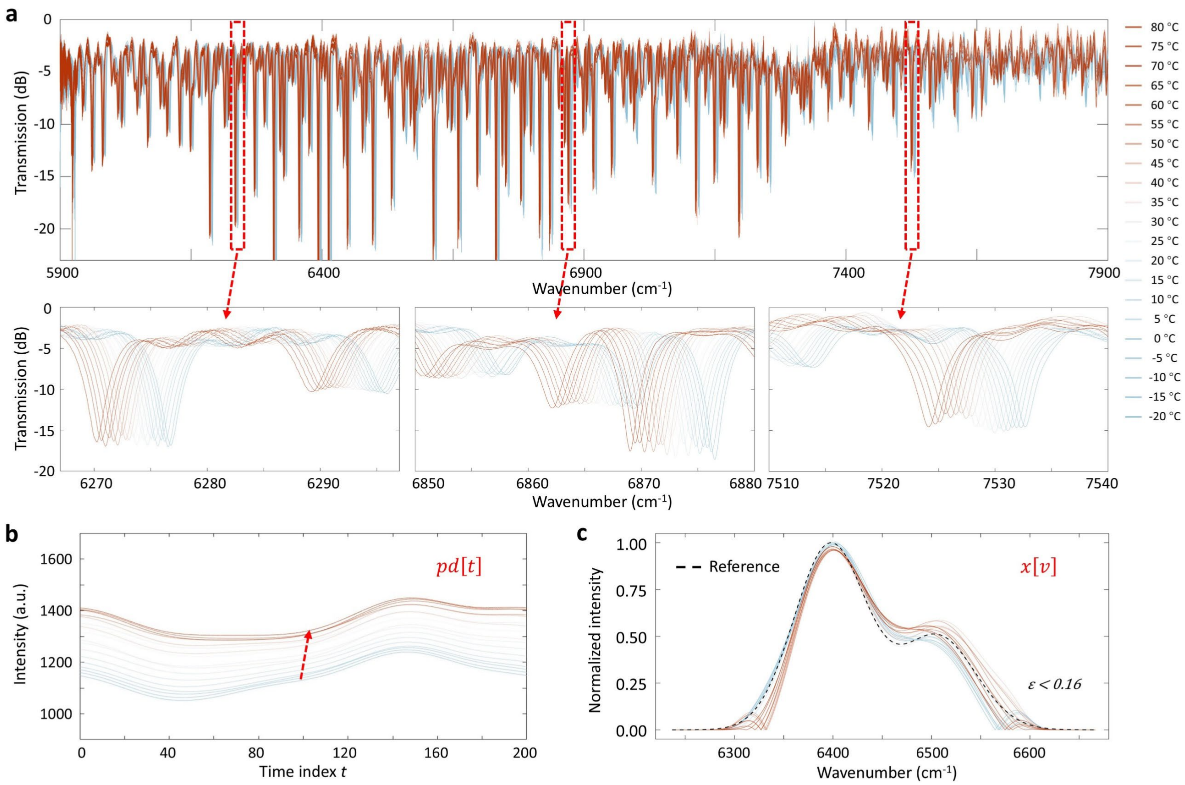

Fig. S8: (a) Measured system responses under ambient temperatures ranging from -20°C to 80°C. The insets show a few representative observation windows, highlighting the consistent waveform shifting caused by the temperature change. (b) Output power intensity sequences for a specific input spectrum under varying ambient temperatures. (c) Resolved spectra after algorithmic correction to counteract the impact of temperature variations, all demonstrating satisfactory accuracy.

implemented to monitor the device's real-time temperature, enabling an algorithmic correction to be applied to the PD sequence to counteract the impact of temperature drift. Figure S8b presents the resolved spectra using this correction scheme across various temperatures, achieving relative errors between 0.02 to 0.16. This demonstrates a superior thermal stability of at-least 100 °C.

Section 8 Optical testbed and sampling interfaces

Figure S9 shows the optical testbed of our ConvSpec, including the calibration of spectral response, convolutional recovery of unknown incident spectra, and various NIR spectrometric sensing demonstrations. During calibration, six SLDs with different center wavelengths are sequentially introduced to the ConvSpec, and the corresponding output spectra are measured using an optical spectral analyzer (YOKOGAWA AQ6370D) to establish the full-band response. Note that this calibration only necessitates recording the initial system response under no phase modulation (see Eq. (2-5)), which requires much less effort compared to those reconstructive spectrometers that must record the entire sampling matrix, i.e., the individual responses of different spatial or temporal sampling channels[6]. While for resolving various unknown input spectra, the output optical power intensities are measured using the co-packaged PD (see Fig. 2b). The recorded power intensity sequences, along with the calibrated system response, are then utilized for spectral recovery based on the convolution theorem.

For the NIR sensing applications, the six SLDs are also sequentially activated to illuminate the samples (i.e. solid and liquid samples or human skin), while the reflected or transmitted light is collected using

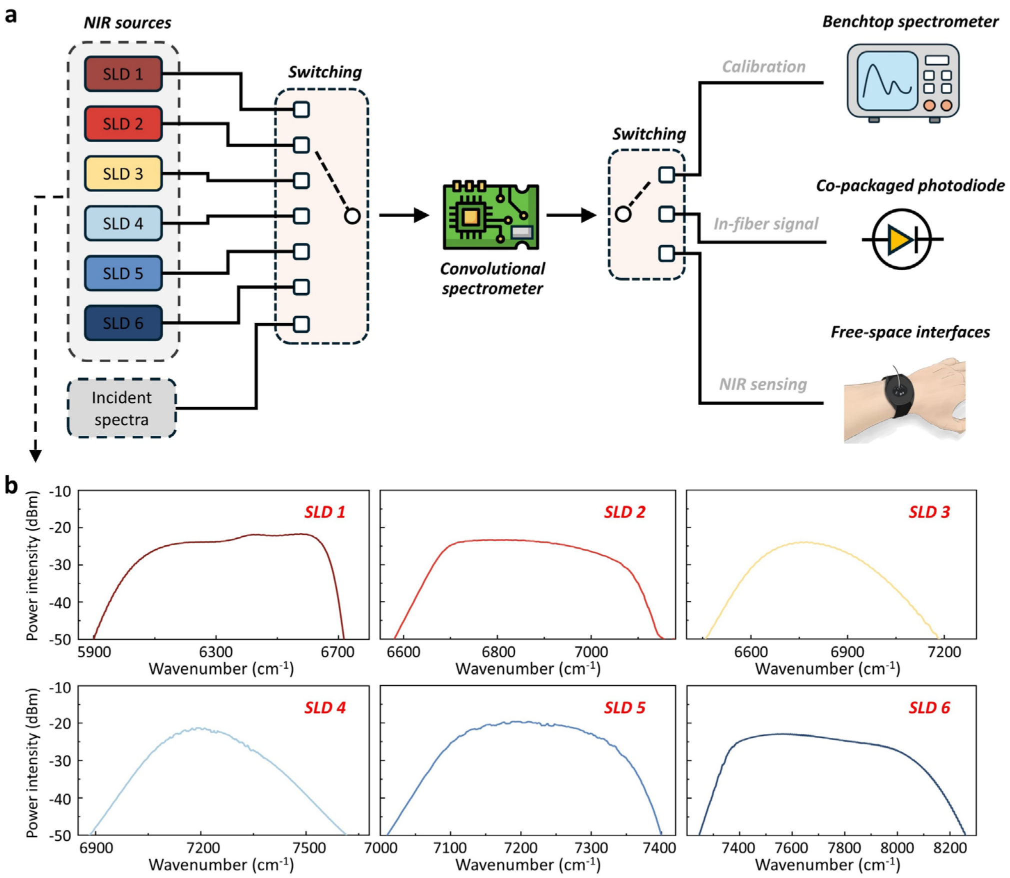

Fig. S9: (a) Schematic of the optical testbed for calibration, unknown spectra recovery, and NIR spectrometric sensing applications. (b) Amplified spontaneous emission (ASE) spectra of the six SLDs.

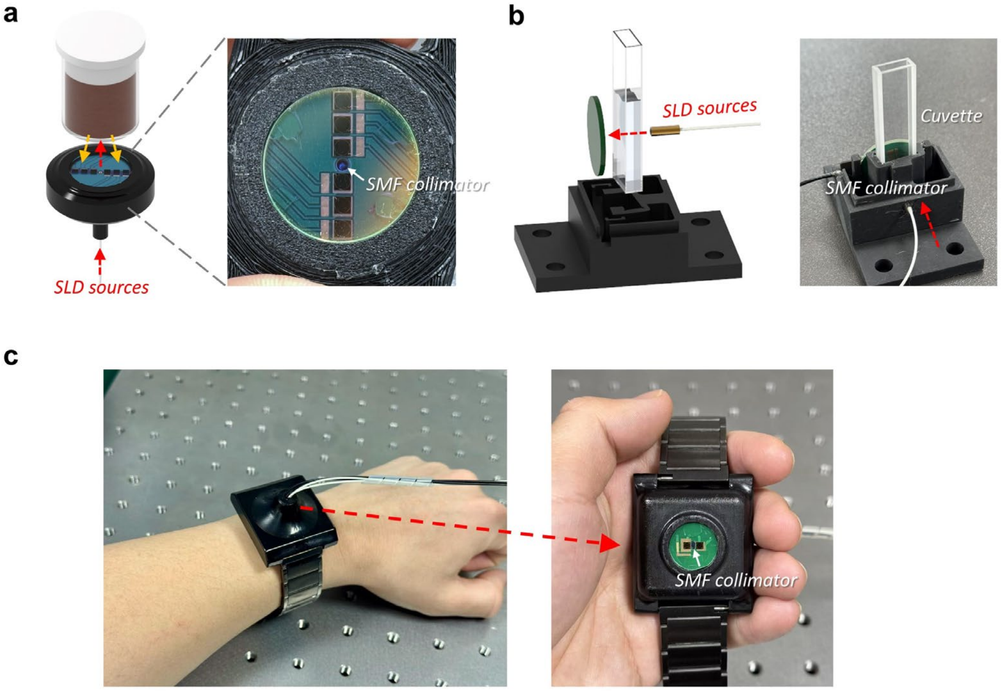

Fig. S10: (a-b) Schematic and photos of the sampling interfaces designed for solid and liquid samples, respectively. (c) Photos of the customized wrist-wearable probe for measuring skin reflectance spectra.

different sampling interfaces. For example, Fig. S10a illustrates the sampling interface designed for solid substances, featuring a single-mode fiber (SMF) lense-based collimator (with a beam diameter of 500 μm) surrounded by 2 mm surface PDs (Suna Optoelectronics, PD-2000-C3). This type of surface PD features a working bandwidth of over 5 MHz, which is far higher than the kHz-level sampling speed required in our experiments. A glass sample container is positioned above this interface, allowing the illuminating SLD sources to reach the sample and then reflect back to the PDs. The measured power intensities are then utilized to calculate the sample reflectance spectra. Likewise, Fig. S10b depicts the sampling interface for liquid samples, which comprises an SMF collimator, a cuvette, and surface PDs. In this setup, the light beam generated by the SLDs transmits through the liquid sample in the cuvette before being collected by the PDs, thereby obtaining the sample's absorptance. For the non-invasive sensing of human biomarkers, a wrist wearable probe is customized to stably measure the skin reflectance spectra, as shown in Fig. S10c. At its center, an SMF collimator directs the SLD illumination onto the skin, while the surrounding PDs capture the reflected light.

Section 9 Detector placement and sensing depth

In skin diffuse-reflectance NIR sensing, the source–detector separation (SDS) is a key parameter that determines both the sampling depth and signal strength. A larger SDS allows photons to travel deeper into tissue but reduces the detected signal, whereas a smaller SDS yields stronger signals dominated by superficial layers. This trade-off is well described by classical diffusion theory and has been confirmed by numerous Monte Carlo and experimental studies[21–23]. Figure S11 shows the typical penetration depth for NIR incident light. Specifically, when the SDS is below 1 mm, the detected signal primarily reflects from

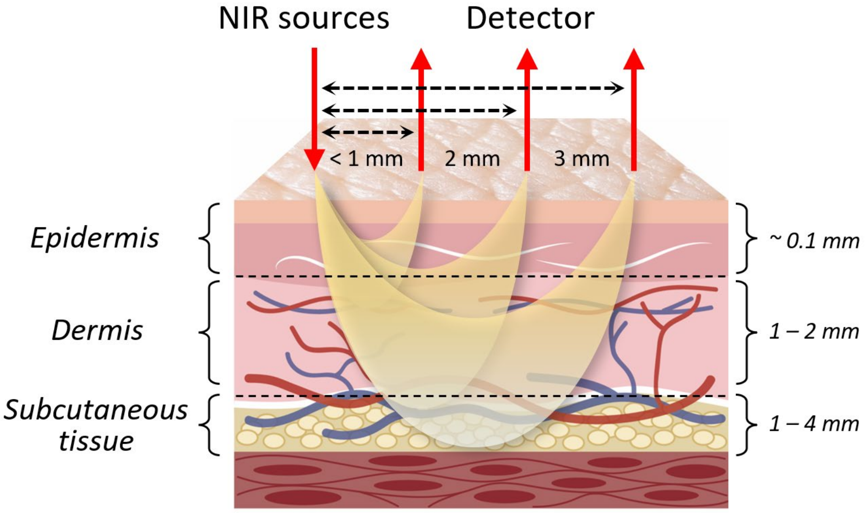

Fig. S11: Schematic illustration of the relationship between source–detector separation (SDS) and tissue sampling depth in skin diffuse-reflectance NIR sensing.

the epidermis and papillary dermis, within an approximate depth of 1 mm. Increasing the SDS to around 2 mm extends the sampling range to include the full dermis and shallow subcutaneous tissue at depths of roughly 1–2 mm. Further increasing the SDS to about 3 mm shifts the sampling depth toward deeper subcutaneous layers, although the signal intensity decreases significantly due to photon attenuation.

In our wearable probe (see Fig. S10c), we employ a surface PD with a 2 mm active area, and the SDS is set to 2 mm, ensuring a good balance between the penetration depth and signal strength. This design is also consistent with previously reported glucose-monitoring probes, which typically use SDS values of 1.5–2.5 mm[24,25].

Section 10 System noise analysis

To quantify the intrinsic noise performance of the proposed ConvSpec system with a wearable probe, we measure its noise floor under both off-body (i.e., static testing) and on-body (i.e., wearable sensing)

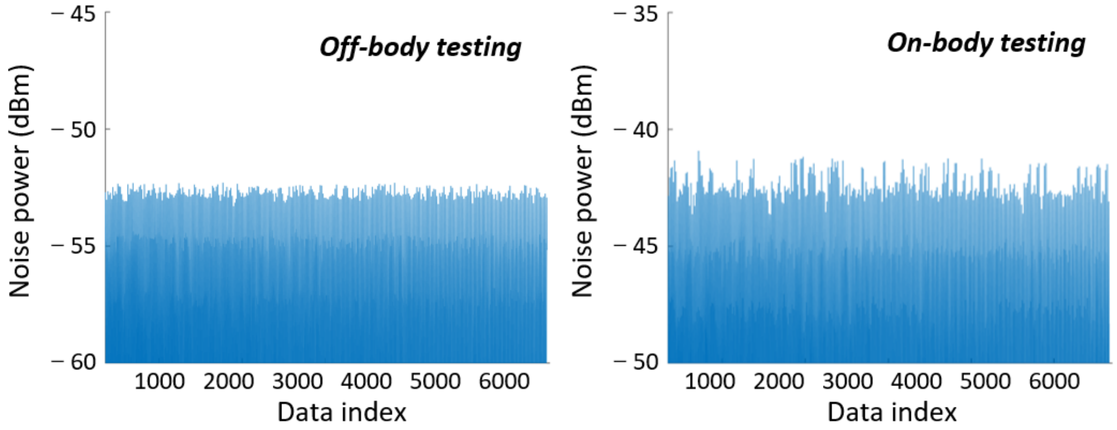

Fig. S12: Measured system noise floor during off-body and on-body measurements, respectively.

conditions, as illustrated in Fig. S12. Under off-body conditions, the measured noise floor is approximately –53 dBm, where the noise primarily originates from the transimpedance amplifier (TIA) circuit, quantization noise of the analogy-to-digital converter (ADC), power supply ripple, and electromagnetic interference (EMI). When the probe is placed on-body, the noise floor increases to approximately –42 dBm. This increase can be attributed to the additional ambient light leakage and micro-motions of the skin.

Given that the aggregated output power of our SLD sources ranges from 0 to +5 dBm and the optical link loss is approximately 16–22 dB (see Fig. 5b), the detected power during our wearable sensing experiments typically falls between –11 dBm and –22 dBm. This corresponds to an effective system signal-to-noise ratio (SNR) of about 20–31 dB during the on-body testing.

Section 11 Additional Spectral and modeling results

Figure S13 presents the reconstruction results of various randomly shaped, continuous spectra generated by a benchtop waveshaper using our ConvSpec. The relative reconstruction errors range from about 0.02 to 0.04, demonstrating the exceptionally high accuracy of our device.

Figure S14 presents the classification results for different pharmaceutical, coffee, flour, and tea samples using the kNN models, all achieving 100% success rates. This highlights the application potential of our spectrometer in industrial, pharmaceutical, and agricultural areas.

Figure S15 presents the predicted concentrations of aqueous ethanol and lactate solutions, as well as organic EG-in-IPA solutions, using SVR models. The calculated MAE values are 0.017%, 0.015%, and 0.006%, while the RMSE values are 0.022%, 0.021%, and 0.013%, respectively.

Figure S16 illustrates the modeling results for predicting glucose solution concentrations with commercial spectrometers (both illuminated with a commercial tungsten-halogen lamp source), using their partial and full working bandwidths for spectral measurement, respectively. In specific, the benchtop

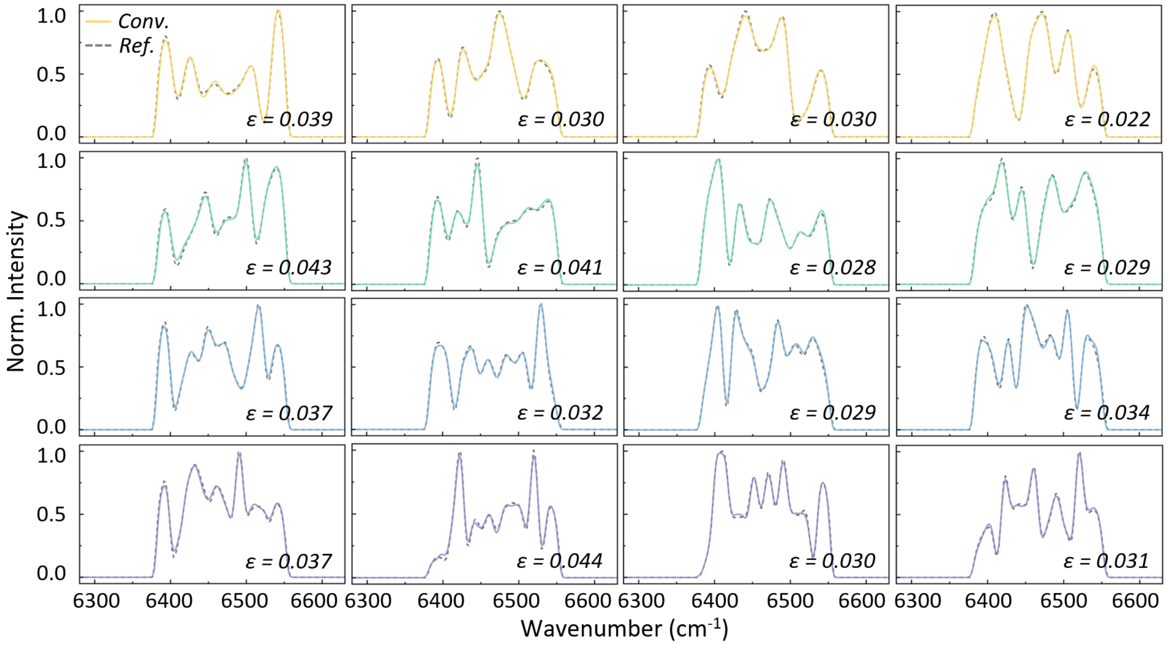

Fig. S13: Resolved randomly shaped, continuous spectra, with waveform complexity increasing progressively from top to bottom row.

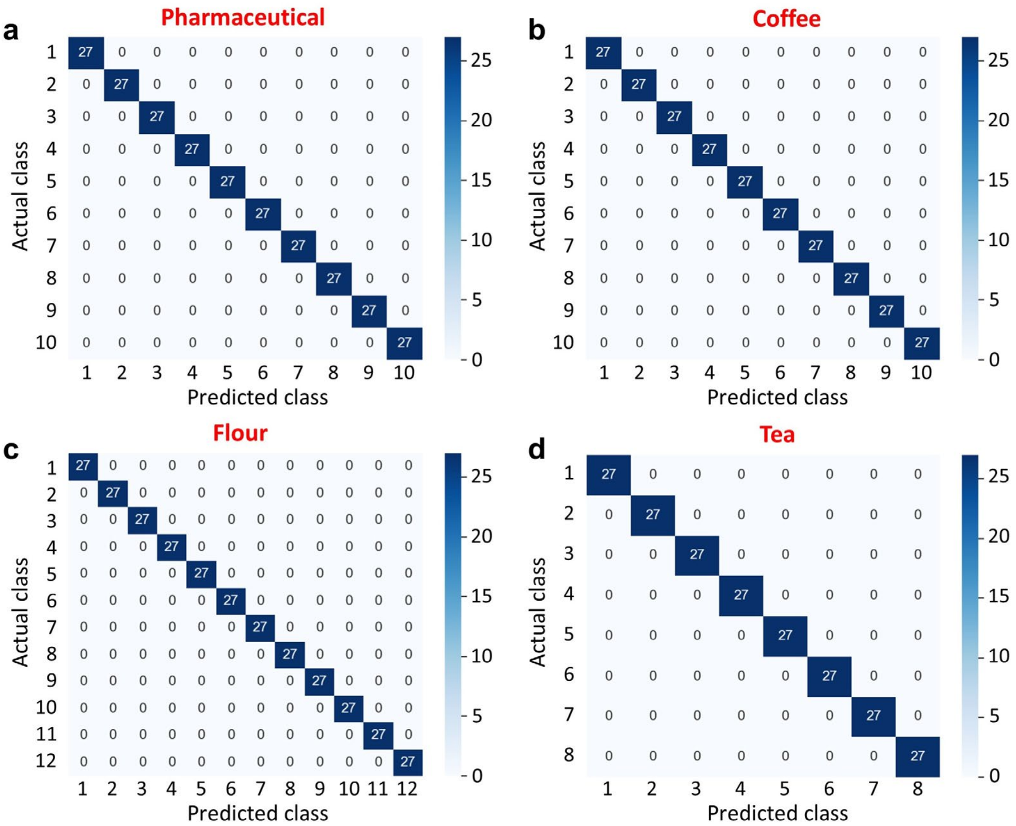

Fig. S14: (a-d) Confusion matrices for the classification results of ten pharmaceutical products, ten coffee varieties, twelve flour types, and eight tea grades, respectively.

Fourier-transform spectrometer (Bruker MPA II) achieves an MAE of 0.009% and an RMSE of 0.012% when utilizing its full bandwidth of 7500 cm^{-1} (from 4000 cm^{-1} to 11500 cm^{-1}). These values slightly degrade to 0.013% and 0.017%, respectively, when the bandwidth is restricted to 2400 cm^{-1} (from 5900 cm^{-1} to 8300 cm^{-1}). In comparison, the dispersive spectrometer (IdeaOptics NIR17+Px) exhibits an MAE of 0.019% and an RMSE of 0.026% with its full bandwidth of 5200 cm^{-1} (from 5850 cm^{-1} to 11050 cm^{-1}). When its bandwidth is also reduced to 2400 cm^{-1}, the performance further declines, with an MAE of 0.026% and an RMSE of 0.034%.

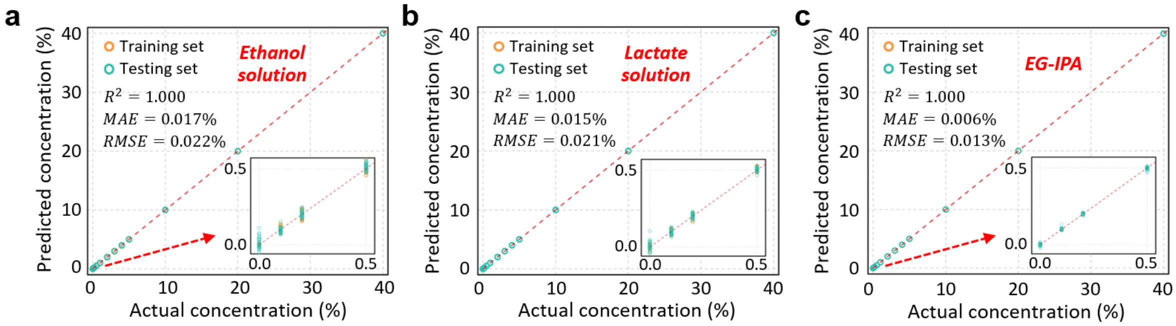

Fig. S15: (a-c) Predicted vs. actual concentrations of ethanol, lactate and EG-in-IPA solutions using SVR models, respectively.

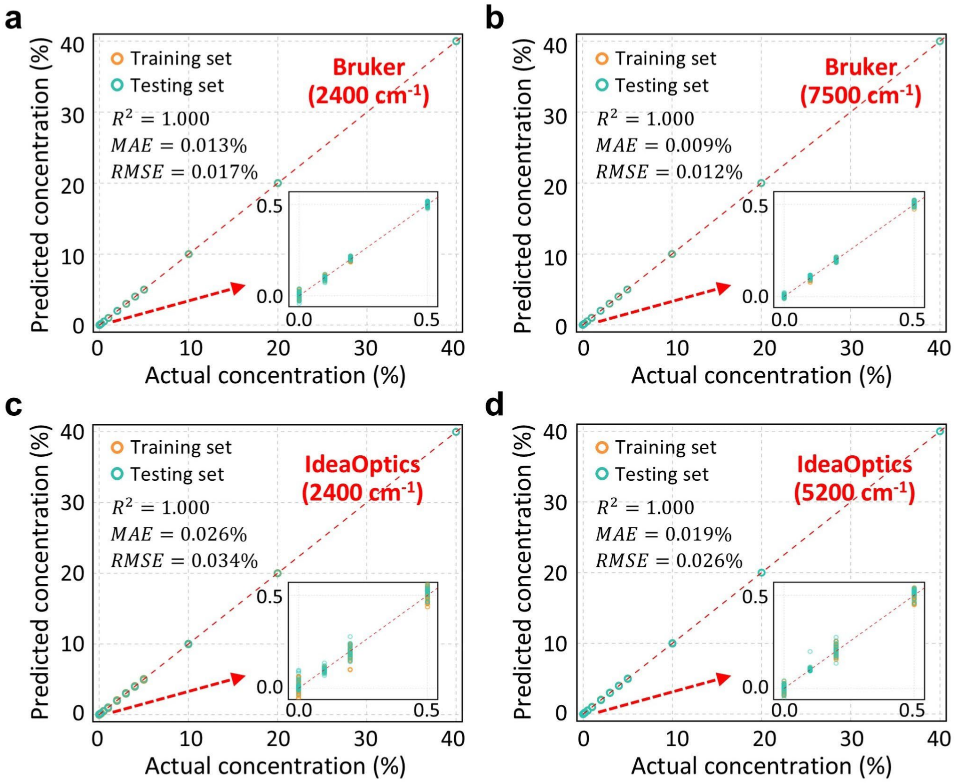

Fig. S16: (a-d) Scatter plots of predicted vs. actual concentrations for glucose solutions measured using different commercial spectrometers with varying operational bandwidths.

Section 12 Statistical summary of participants

Table S2 provides a statistical summary of the number of participants, gender distribution, and age groups for the testing of skin moisture, blood alcohol, blood lactate, and blood glucose (including the single-

Table S2. Statistical summary of participants

Biomarker	Number of participants	Participant gender	Age distribution				Total spectra collected
			< 25 yrs	25-30 yrs	30-35 yrs	> 35 yrs	
Skin moisture	26	Male	5	4	4	2	1068
		Female	3	4	2	2	
Blood alcohol	24	Male	6	3	0	3	1056
		Female	3	4	2	3	
Blood lactate	48	Male	9	12	0	3	1152
		Female	6	9	6	3	
Blood glucose	27	Male	3	3	3	3	1107
		Female	3	3	6	3	
Blood glucose (long-term)	1	Male - 21 yrs					1380

participant long-term trial), respectively. In total, our study comprises n = 126 participant sessions and collects around 6,000 full-band human spectra.

Section 13 Additional data on biomarker sensing experiments

Figure S17 presents the distribution of prediction errors in the SVR modeling for skin moisture, blood alcohol, blood lactate, and blood glucose, respectively (corresponding to Fig. 5d, 5f, 5h, and 5j in the main text). The modeling MAEs are 2.45%, 1.70 mg dL^{-1}, 0.81 mmol L^{-1} and 0.36 mmol L^{-1}, while the 80th percentiles of errors are 3.98%, 2.59 mg dL^{-1}, 1.28 mmol L^{-1} and 0.574 mmol L^{-1}, respectively.

On the other hand, Figure S18a presents the raw and preprocessed spectra collected during the skin moisture testing. Figures S18b–d further display the preprocessed spectra measured from all participants during the testing of blood alcohol, blood lactate, and blood glucose, respectively.

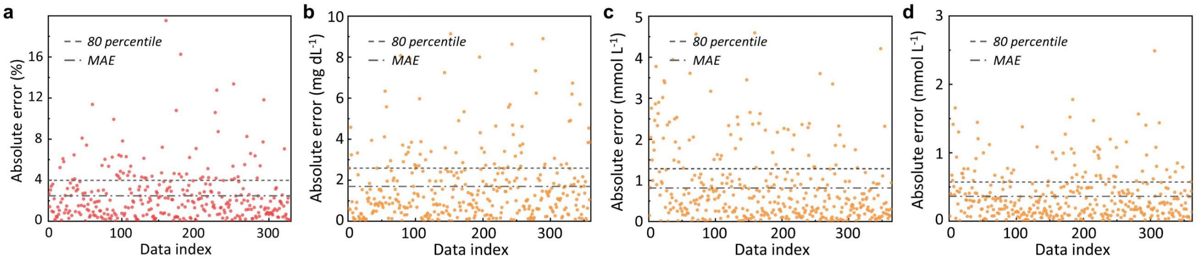

Fig. S17: (a-d) Distribution of prediction errors in the SVR modeling of spectra collected during the skin moisture, blood alcohol, blood lactate, and blood glucose experiments, respectively.

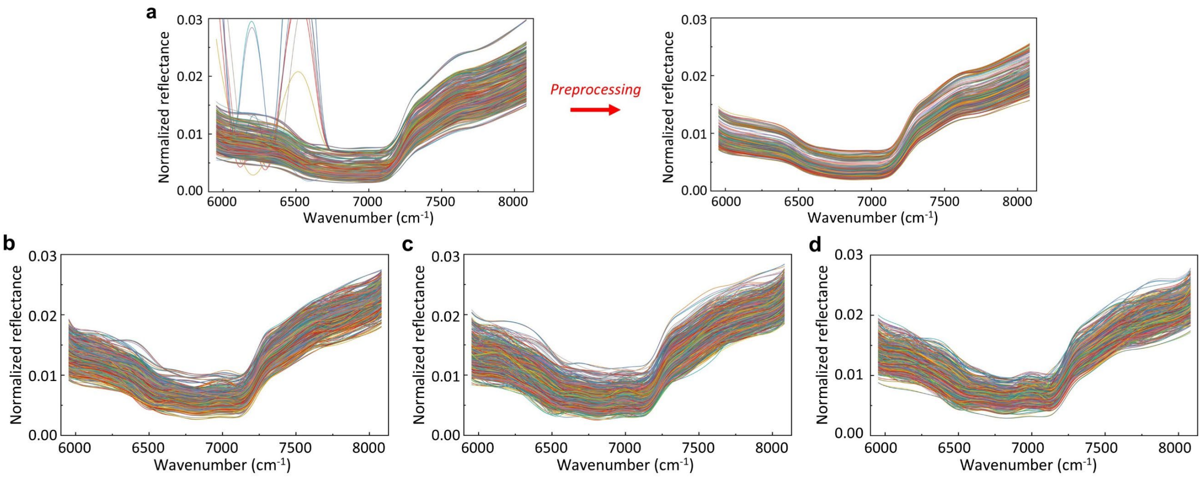

Fig. S18: (a) Raw and preprocessed reflectance spectra measured at varying skin moisture levels for different participants, respectively. (b-d) Measured (preprocessed) spectra from participants across varying levels of blood alcohol, lactate, and glucose, respectively.

Section 14 Incorporating heart rate in blood lactate modeling

Given that heart rate is strongly correlated with exercise intensity, we further investigate the effect of incorporating participants' real-time heart rates as an additional data dimension in the modeling of blood lactate concentration. Figure S19 presents the modeling results under this condition, achieving an MAE of 0.454 mmol L^{-1} and an RMSE of 0.669 mmol L^{-1}. This corresponds to an almost twofold reduction in error

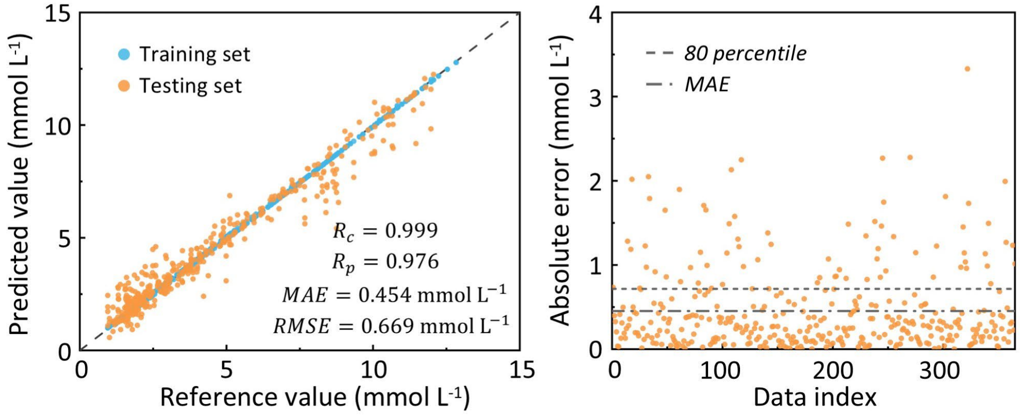

Fig. S19: Predicted and reference blood lactate concentrations using the SVR model that incorporated participants' real-time heart rates (left). The distribution of absolute measurement errors (right).

compared to modeling based solely on spectral data (i.e. 0.814 mmol L^{-1} and 1.201 mmol L^{-1}, respectively). Thus, for certain biomarkers, it could be beneficial to integrate relevant physiological variables to further enhance the model accuracy.

Section 15 Additional discussions on the blood glucose model

In the main text, we have demonstrated the transferability of our skin moisture model, showing that a model pre-trained on spectral data from other participants can accurately predict the skin moisture levels of a new participant (see Fig. 5e). This success is attributed to not only the measurement precision and stability of our spectrometer, but also to the relatively prominent spectral features of water molecules on the skin surface. However, for subsurface blood metabolites, particularly blood glucose, the situation is far more complex. The inherently low concentration of blood glucose results in weak spectral signals that can be easily obscured by inter-individual variations. Here, we discuss how the selection of training datasets affects

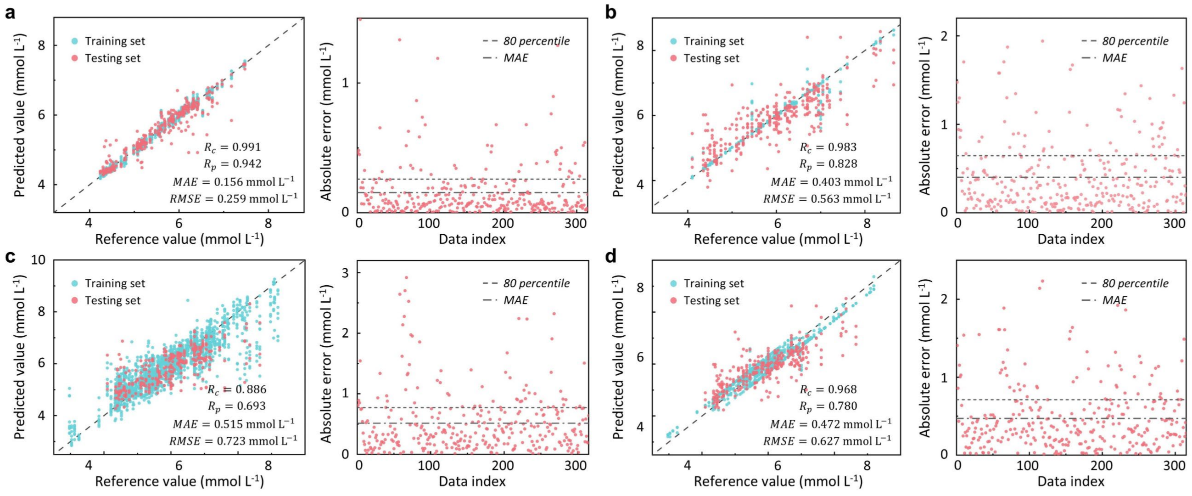

Fig. S20: (a-d) Predicted and reference concentrations of blood glucose using the single-participant DFNN model, single-participant SVR model, mixed-source DFNN model, and mixed-source SVR model, along with their respective distributions of absolute measurement errors.

the model accuracy in glucose concentration prediction. Figures S20a-b illustrate the modeling performance using spectral data from a single participant in our long-term glucose monitoring experiment, applying the DFNN and SVR algorithms, respectively (i.e. Fig. S20a replicates Fig. 5l in the main text). Overall, both algorithms effectively extract glucose-related information in single-participant modeling, with DFNN providing better model correlation and accuracy. However, when we introduce additional spectral data from the multi-participant OGTT experiment into the training set, i.e. creating a mixed-source dataset that incorporates inter-individual variability while still retaining the target single participant, the correlation and prediction accuracy exhibit a noticeable decline, as shown by Fig. S20c-d. Moreover, if we entirely exclude the single participant's data and train the model solely on multi-participant data, the model fails to establish any meaningful correlation or predictive accuracy. This, unfortunately, indicates that at our current dataset scale, the model is still unable to achieve effective cross-participant transferability. This remains a common challenge in the field, requiring a significant expansion of the dataset and the adoption of more advanced algorithms to overcome.

Section 16 Review of miniaturized spectrometers and NIR spectroscopy studies

To highlight the performance advantages of our ConvSpec, we summarize the key performance metrics of the state-of-the-art miniaturized spectrometers based on different working principles and material platforms, as listed in Table S3. The results affirm our superiority not only in bandwidth, spectral accuracy, and sampling and computing speed, but also in packaging level and large-scale manufacturability.

Table S4 further reviews a broad range of NIR spectroscopy studies across different application scenarios, from material analysis to biomedical sensing, utilizing various types of spectrometers. It can be observed that mainstream NIR studies still predominantly rely on bulky and expensive commercial spectrometers, with only a few employing prototype devices for proof-of-concept validation. In comparison, our ConvSpec offers overwhelming advantages in size and cost, while also delivering bandwidth, resolution, and modeling accuracy that are comparable to those of commercial products.

Table S3. Performance comparison against the state-of-the-art miniaturized spectrometers

Working principle & material	BW.	RES.	Spectral accuracy	Sampling/ computing speed	Packaging level	Device (chip) size	CMOS-compatible
AWG (dispersive); SOI[26]	10 nm	0.1 nm	N.M.	N.A.	Bare die	8 × 8 mm^2	Yes
EDG (dispersive); SOI[27]	24 nm	0.5 nm	N.M.	N.A.	Bare die with integrated PD	6 × 9 mm^2	Yes
Holographic planar circuit (dispersive) ; SiN[28]	148 nm	0.15 nm	N.M.	N.A.	Bare die	200 mm^2	No
Microdonut resonator (narrowband filtering); SOI[29]	50 nm	0.6 nm	N.M.	N.A.	Bare die	1 mm^2	Yes
Tunable MRR (narrowband filtering); SOI[30]	10 nm	5 pm	N.M.	1.7 s sampling	Bare die	0.35 mm^2	Yes

MRR + AWG (narrowband filter), SOI[31]	70 nm	0.2 nm	N.M.	1.1 ms sampling (theoretical)	Bare die with integrated Ge PD	0.270 × 0.2 mm^2 (AWG)	Yes
Tunable F-P cavity (narrowband filter); SOI[32]	103 nm	0.51 nm	N.M.	0.35 s sampling	Bare die	43 × 840 μm^2	Yes
Cascaded MRRs (narrowband filter); SOI[33]	230 nm	30 pm	N.M.	N.M.	Wire-bonded	0.34 mm^2	Yes
MZI array (Fourier transform); SOI[34]	0.75 nm	0.04 nm	N.M.	N.A.	Bare die	12 mm^2	Yes
Tunable MZI (Fourier transform); SOI[14]	233.5 cm^{-1}	12.7 cm^{-1}	N.M.	N.M.	Bare die	1 mm^2	Yes
MZI array (Fourier transform); SiN[35]	340 nm	49 pm	N.M.	N.A.	Bare die	9.8 × 0.9 mm^2	Yes
MZI + switch (Fourier transform); SOI[36]	20 nm	0.2 nm	$R^2 > 0.82$	2.7 s sampling	Fully packaged + integrated Ge PD	N.M.	Yes
MZI + MRR (Fourier transform); SOI[15]	90 nm	0.47 nm	N.M.	2 s sampling	Wire-bonded + integrated Ge PD	N.M.	Yes
Dual-layer MZI (Fourier transform); dual-layer SiN[37]	7 nm	0.4 nm	$R^2 > 0.95$	N.A.	Fiber-attached	5.8 × 4.6 mm^2	No
Stationary wave (Fourier transform); SiN[38]	100 nm	6 nm	N.M.	N.A.	Bare die	0.1 mm^2	Yes
Nanowire (reconstructive), CdS_xSe_{1-x} nanowire[39]	130 nm	10 nm	N.M.	N.M.	Wire-bonded	0.5 × 75 μm^2	No
Van der Waals junction (reconstructive), MoS_2/WSe_2 heterojunction[40]	440 nm	3 nm	N.M.	N.M.	Wire-bonded	22 × 8 μm^2	No
Van der Waals junction (with memory) (reconstructive), $SnS_2/ReSe_2$ heterojunction[41]	400 nm	5 nm	N.M.	40 s sampling	N.M.	220 μm^2	No
Quantum dot (reconstructive); SiN[42]	300 nm	2 nm	N.M.	N.A.	Bare die	8.5 × 6.8 mm^2	No

Coherent network (reconstructive); CdS and CdSe[43]	12 nm	0.02 nm	ε < 0.0438	N.A.	Bare die	520 × 220 μm^2	Yes
Disordered media (reconstructive), SiN+polymer[44]	40 nm	3.4 nm	N.M.	N.A.	Optical wire-bonded	N.M.	No
Multi-resonant cavities (reconstructive); SiN[45]	270 nm	0.5 nm	ε < 0.133	N.A.	Bare die	N.M.	Yes
Multi-mode cavity (reconstructive); SOI[46]	100 nm	5 pm	ε < 0.18, $R^2 > 0.98$	0.2 s sampling	Wire-bonded	1 × 1.5 mm^2	Yes
Microdisk resonator (reconstructive); SOI[47]	20 nm	0.2 nm	ε = 0.029	3 s computing	Bare die	200 × 200 μm^2	Yes
Reconfigurable network (reconstructive); SiN[6]	115 nm	0.03 nm	ε < 0.083	0.4s sampling + > 9 s computing	Wire-bonded	2.0 × 7.6 mm^2	Yes
Programmable circuits (reconstructive); SiN[48]	200 nm	0.01 nm	ε < 0.074	0.7 s sampling + > 70 s computing	Wire-bonded	1.9 × 3.7 mm^2	Yes
MRR + TPA PD (reconstructive); SOI[49]	10 nm	0.4 nm	ε < 0.70	N.M.	Wire-bonded, integrated TPA PD	16 × 16 μm^2 (MRR)	Yes
Metasurface+CMOS (reconstructive); SOI[50]	100 nm	70 pm	ε < 0.45	N.A.	N.M.	0.15 × 0.95 mm^2	Yes
Chaos-assited MRRs (reconstructive); SOI[51]	100 nm	10 pm	ε < 0.22	N.M.	Wire-bonded	20 × 22 μm^2 (MRR)	Yes
Cascaded MRRs (reconstructive); SiN[52]	520 nm	8 pm	ε < 0.11	4 s sampling + > 500 s computing	Fully packaged	80 × 150 μm^2 per MRR	Yes
This work: Convolutional spectrometer; SiN	2400 cm^{-1} (i.e. 500 nm)	5.8 cm^{-1} (i.e. 1.4 nm)	ε < 0.044	0.3 s sampling + 50 ms computing	Fully packaged	0.9 × 3.4 mm^2	Yes

*BW.: bandwidth; Res.: resolution; N.A.: not applicable; N.M.: not mentioned; SOI: Silicon-on-isolator; AWG: arrayed waveguide grating; EDF: Echelle diffraction grating; F-P: Fabry-Pérot; TPA: two-photon absorption.

Table S4. Review of NIR spectroscopic studies

**Application scenarios & measurement scheme**	**Spectrometer module**	**Size & Cost**	**BW.**	**RES.**	**Model algorithm & performance**

Quality monitoring of cow's milk (ex-situ)[53]	FOSS NIR Systems 6500 (commercial; dispersive)	Benchtop 63×38×30 cm^3 >$2,800	400 nm – 2500 nm	2 nm	PLS regression with R^2 of > 0.810
Composition analysis of instant tea (ex-situ)[54]	Hitachi U-3010 UV-visible (commercial; dispersive)	Benchtop 68×69×26 cm^3 >$1,000	1000 nm – 2500 nm	2 nm	BPSO and SVR with R_p of > 0.757
Sugar quantification in milk tablets (ex-situ)[55]	FOSS NIR Systems 6500 (commercial; dispersive)	Benchtop 63×38×30 cm^3 >$2,800	400 nm – 2500 nm	2 nm	PLS regression with R^2 of > 0.975
Predicting maca power adulteration (ex-situ)[56]	DLP NIR Scan Nano (commercial; dispersive)	Portable 62×58×36 mm^3 >$1,750	900 nm – 1700 nm	8 nm	LDA and PLS regression with R^2 of > 0.910
Land cover classification (in-situ)[57]	Flame-NIR Miniature (commercial; dispersive)	Portable 89×63×32 mm^3 >$10,000	950 nm – 1650 nm	10 nm	Minimum distance classifier with accuracy of 78%
Plastic classification and quantification of solution concentration (ex-situ)[52]	SiN spectrometer chip (prototype; reconstructive)	Portable 70×60×20 mm^3 N.M.	1200 nm – 1700 nm	N.M.	SVR with 100% accuracy, and RF with R^2 of 1.00
Serum analysis for discriminating breast cancer (in-vitro)[58]	Bruker Tensor 27 (commercial; Fourier transform)	Benchtop 67×43×28 cm^3 >$59,000	370 cm^{-1} – 7500 cm^{-1}	1 cm^{-1}	LDA with an accuracy of 90%
Measuring the content of serum albumin (in-vitro)[59]	XDS Rapid Content™ (commercial; dispersive)	Benchtop 46×57×39 cm^3 >$10,000	400 nm – 2500 nm	2 nm	PLS regression with R_p of > 0.997
Plasma analysis for glucose, triglycerides, urea, and cholesterol (in-vitro)[60]	Bruker Tensor 27 (commercial; Fourier transform)	Benchtop 67×43×28 cm^3 >$59,000	370 cm^{-1} – 7500 cm^{-1}	1 cm^{-1}	Deming linear regression with R^2 of > 0.980
Hemodialysis for urea, glucose, lactate, phosphate, and creatinine (in-vitro)[61]	NIRFlex N-500 (commercial; Fourier transform)	Benchtop 35×25×45 cm^3 >$2,000	4000 cm^{-1}– 12500 cm^{-1}	8 cm^{-1}	PLS regression with R^2 of > 0.860
Non-invasive glucose analysis for rats (in-vivo)[62]	Bruker Matrix-F FT-NIR (commercial; Fourier transform)	Benchtop 48×31×23 cm^3 >$10,000	4000 cm^{-1}– 12000 cm^{-1}	8 cm^{-1}	PLS and ANN with R^2 of 0.962 and 0.928
Non-invasive oral glucose test on inner lip (in-vivo)[63]	Bruker IFS-66 (commercial; Fourier transform)	Benchtop N.M. >$15,00	4000 cm^{-1}– 10000 cm^{-1}	32 cm^{-1}	Multivariate calibration with MARD of 23%
Non-invasive sensing of blood glucose (in-vivo)[64]	DLP NIRscan Nano EVM (commercial; dispersive)	Portable 11×6×8 cm^3 >$10,50	900 nm – 1700 nm	10 nm	PLS regression with R_p of 0.958
Non-invasive sensing of blood glucose (in-vivo)[65]	Handmade optical system (prototype; direct intensity measurement)	Portable N.M. N.M.	940 & 1300 nm	N.A.	Huber's regression model with R^2 of 0.908

Non-invasive sensing of blood glucose (in-vivo)[23]	Handmade optical sensor (prototype; direct intensity measurement)	Portable N.M. N.M.	1050, 1219,1314, 1380,1550, &1609 nm	N.A.	PLS regression with average R_p of 0.790
This work: Solid classification, solution quantification, and non-invasive sensing of skin moisture, blood alcohol, lactate and glucose (in-vivo)	SiN spectrometer chip (prototype; convolutional)	Portable 4.8×6×0.6 cm^3 ~$10	5900 cm^{-1}– 8300 cm^{-1}	5.8 cm^{-1}	kNN with 100% accuracy; SVR with R^2 of 1.000; SVR with R_p of > 0.919

*BW.: bandwidth; RES.: resolution; N.A.: not applicable; N.M.: not mentioned. R^2: coefficient of determination; R_p: prediction correlation coefficient; PLS: partial least square; BPSO: binary particle swarm optimization; SVR: support vector regression; LDA: linear discriminant analysis; RF: random forest; ANN: artificial neural networks; kNN: k-nearest neighbors; MARD: mean absolute relative difference.

Section 17 Cross-validation between models

To demonstrate the independence of our biomarker models, we conduct cross-validation between the blood glucose and lactate models as a representative example by testing each dataset with the other's model, as shown in Fig. S21. The resulting prediction correlation coefficients R_p are −0.020 and −0.034, respectively, indicating no correlation between the two. This confirms that our glucose and lactate models extract distinct features specific to their respective biomarkers, ensuring a single-contributor characteristic.

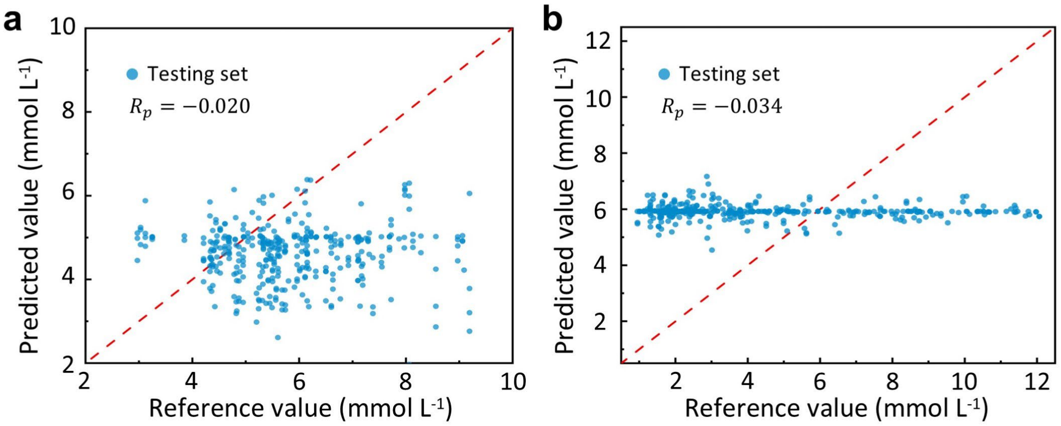

Fig. S21: (a) Prediction of blood glucose concentrations using the lactate model. (b) Prediction of blood lactate concentrations using the glucose model.

Section 18 Modeling performance across different algorithms

Here, we use the multi-participant testing of blood glucose as a representative example to evaluate the modeling performance across different algorithms. We examine a range of regression algorithms, from basic regression methods such as linear regression and Ridge regression to mainstream machine learning approaches including SVR, random forests, gradient boosting trees, extreme gradient boosting (XGBoost), light gradient boosting machine (LightGBM), and CatBoost (a gradient boosting algorithm optimized for categorical feature processing). Additionally, we test various neural network architectures, including

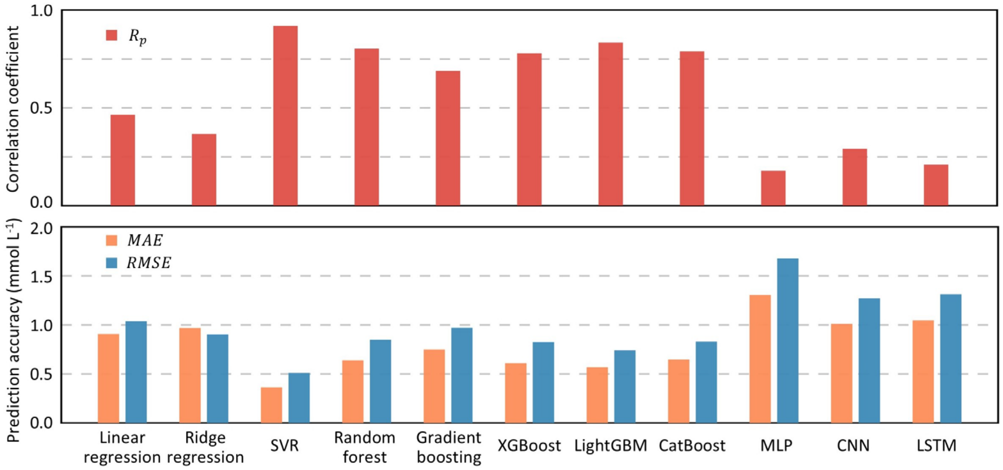

Fig. S22: Prediction correlation coefficient (top), as well as MAE and RMSE (bottom), of multi-participant blood glucose models based on different algorithms, respectively.

multilayer perceptron (MLP), convolutional neural network (CNN), and long short-term memory (LSTM; a variant of the recurrent neural network, RNN). Figure S22 illustrates the prediction correlation coefficient R_p, MAE and RMSE achieved by different algorithms. The results suggest that machine learning algorithms, particularly SVR, demonstrate stronger model correlation and lower prediction errors, whereas neural networks tend to underperform. This can be attributed to the fact that neural networks require a sufficiently large training set to effectively extract meaningful spectral features while mitigating the impact of inter-individual variations.

Reference

1. Saadany, B. *et al.* MEMS tunable Michelson interferometer with robust beam splitting architecture. in *2009 IEEE/LEOS International Conference on Optical MEMS and Nanophotonics* 49–50 (2009).
2. Shaheen, A. K., Sabry, Y. M. & Khalil, D. A. Combined MEMS spectrometer based on Michelson interferometer and tunable filter for wideband selective operation. in *MOEMS and Miniaturized Systems XIX* vol. 11293 111–118 (SPIE, 2020).
3. Hill, K. O. & Meltz, G. Fiber Bragg grating technology fundamentals and overview. *Journal of lightwave technology* **15**, 1263–1276 (1997).
4. James, S. W. & Tatam, R. P. Optical fibre long-period grating sensors: characteristics and application. *Measurement science and technology* **14**, R49 (2003).
5. Nussbaum, P., Voelkel, R., Herzig, H. P., Eisner, M. & Haselbeck, S. Design, fabrication and testing of microlens arrays for sensors and microsystems. *Pure and applied optics: Journal of the European optical society part A* **6**, 617 (1997).
6. Yao, C. *et al.* Broadband picometer-scale resolution on-chip spectrometer with reconfigurable photonics. *Light Sci Appl* **12**, 156 (2023).
7. Su, Y., Zhang, Y., Qiu, C., Guo, X. & Sun, L. Silicon Photonic Platform for Passive Waveguide Devices: Materials, Fabrication, and Applications. *Adv. Mater. Technol.* **5**, 1901153 (2020).
8. Li, Z. *et al.* High density lithium niobate photonic integrated circuits. *Nat Commun* **14**, 4856 (2023).
9. Yan, Z. *et al.* A monolithic InP/SOI platform for integrated photonics. *Light Sci Appl* **10**, 200 (2021).

10. Keyvaninia, S. *et al.* Demonstration of a heterogeneously integrated III-V/SOI single wavelength tunable laser. *Opt. Express, OE* **21**, 3784–3792 (2013).
11. Vaseghi, S. V. *Advanced Digital Signal Processing and Noise Reduction*. (John Wiley & Sons, 2008).
12. Ilesanmi, A. E. & Ilesanmi, T. O. Methods for image denoising using convolutional neural network: a review. *Complex Intell. Syst.* **7**, 2179–2198 (2021).
13. Gurov, I., Hlubina, P. & Chugunov, V. Evaluation of spectral modulated interferograms using a Fourier transform and the iterative phase-locked loop method. *Meas. Sci. Technol.* **14**, 122 (2002).
14. Souza, M. C. M. M., Grieco, A., Frateschi, N. C. & Fainman, Y. Fourier transform spectrometer on silicon with thermo-optic non-linearity and dispersion correction. *Nat Commun* **9**, 665 (2018).
15. Zheng, S. N. *et al.* Microring resonator-assisted Fourier transform spectrometer with enhanced resolution and large bandwidth in single chip solution. *Nat Commun* **10**, 2349 (2019).
16. Platform – Advanced Micro Foundry. https://www.advmf.com/platform/.
17. Our Services - CORNERSTONE | Technology platforms. *CORNERSTONE* https://www.cornerstone.sotonfab.co.uk/our-services/.
18. Morino, H., Maruyama, T. & Iiyama, K. Reduction of Wavelength Dependence of Coupling Characteristics Using Si Optical Waveguide Curved Directional Coupler. *J. Lightwave Technol., JLT* **32**, 2188–2192 (2014).
19. Yao, C., Cheng, Q., Roelkens, G. & Penty, R. Bridging the gap between resonance and adiabaticity: a compact and highly tolerant vertical coupling structure. *Photon. Res., PRJ* **10**, 2081–2090 (2022).
20. Milanizadeh, M., Aguiar, D., Melloni, A. & Morichetti, F. Canceling Thermal Cross-Talk Effects in Photonic Integrated Circuits. *J. Lightwave Technol., JLT* **37**, 1325–1332 (2019).
21. Farrell, T. J., Patterson, M. S. & Wilson, B. A diffusion theory model of spatially resolved, steady-state diffuse reflectance for the noninvasive determination of tissue optical properties in vivo. *Medical physics* **19**, 879–888 (1992).
22. Bashkatov, A. N., Genina, E. A., Kochubey, V. I. & Tuchin, V. Optical properties of human skin, subcutaneous and mucous tissues in the wavelength range from 400 to 2000 nm. *Journal of Physics D: Applied Physics* **38**, 2543 (2005).
23. Ge, Q. *et al.* Evaluation and validation on sensitivity of near-infrared diffuse reflectance in non-invasive human blood glucose measurement. *Sensors* **24**, 5879 (2024).
24. Xu, K., Qiu, Q., Wang, W. & Jiang, J. The interface between probe and skin in noninvasive glucose sensing. in vol. 5068 104–111 (SPIE, 2003).
25. Chatterjee, S., Budidha, K., Qassem, M. & Kyriacou, P. A. In-silico investigation towards the non-invasive optical detection of blood lactate. *Scientific Reports* **11**, 14274 (2021).
26. Cheben, P. *et al.* A high-resolution silicon-on-insulator arrayed waveguide grating microspectrometer with sub-micrometer aperture waveguides. *Optics express* **15**, 2299–2306 (2007).
27. Ma, X., Li, M. & He, J.-J. CMOS-Compatible Integrated Spectrometer Based on Echelle Diffraction Grating and MSM Photodetector Array. *IEEE Photonics Journal* **5**, 6600807–6600807 (2013).
28. Calafiore, G. *et al.* Holographic planar lightwave circuit for on-chip spectroscopy. *Light: Science & Applications* **3**, e203–e203 (2014).
29. Xia, Z. *et al.* High resolution on-chip spectroscopy based on miniaturized microdonut resonators. *Optics express* **19**, 12356–12364 (2011).
30. Zhang, L. *et al.* Ultrahigh-resolution on-chip spectrometer with silicon photonic resonators. *Opto-Electronic Advances* **5**, 210100–1 (2022).

31. Zhang, Z. *et al.* Integrated scanning spectrometer with a tunable micro-ring resonator and an arrayed waveguide grating. *Photon. Res., PRJ* **10**, A74–A81 (2022).
32. Sun, C. *et al.* Broadband and High-Resolution Integrated Spectrometer Based on a Tunable FSR-Free Optical Filter Array. *ACS Photonics* **9**, 2973–2980 (2022).
33. Lv, D. *et al.* High-Resolution and Broad-Band On-Chip Optical Spectrometer Beyond 1.55 μm. *Laser & Photonics Reviews*, e00294.
34. Velasco, A. V. *et al.* High-resolution Fourier-transform spectrometer chip with microphotonic silicon spiral waveguides. *Opt. Lett.* **38**, 706 (2013).
35. González-Andrade, D. *et al.* Broadband Fourier-transform silicon nitride spectrometer with wide-area multiaperture input. *Opt. Lett., OL* **46**, 4021–4024 (2021).
36. Kita, D. M. *et al.* High-performance and scalable on-chip digital Fourier transform spectroscopy. *Nat Commun* **9**, 4405 (2018).
37. Lu, L., Zhang, H., Li, X., Chen, J. & Zhou, L. Low temperature sensitivity on-chip Fourier-transform spectrometer based on dual-layer Si_3N_4 spiral waveguides. *Photon. Res., PRJ* **11**, 591–599 (2023).
38. Nie, X., Ryckeboer, E., Roelkens, G. & Baets, R. CMOS-compatible broadband co-propagative stationary Fourier transform spectrometer integrated on a silicon nitride photonics platform. *Opt. Express, OE* **25**, A409–A418 (2017).
39. Yang, Z. *et al.* Single-nanowire spectrometers. *Science* **365**, 1017–1020 (2019).
40. Yoon, H. H. *et al.* Miniaturized spectrometers with a tunable van der Waals junction. *Science* **378**, 296–299 (2022).
41. Wu, G. *et al.* Miniaturized spectrometer with intrinsic long-term image memory. *Nat Commun* **15**, 676 (2024).
42. Bao, J. & Bawendi, M. G. A colloidal quantum dot spectrometer. *Nature* **523**, 67–70 (2015).
43. Zhang, Z. *et al.* Compact High Resolution Speckle Spectrometer by Using Linear Coherent Integrated Network on Silicon Nitride Platform at 776 nm. *Laser & Photonics Reviews* 2100039 (2021).
44. Hartmann, W. *et al.* Waveguide-Integrated Broadband Spectrometer Based on Tailored Disorder. *Advanced Optical Materials* **8**, 1901602 (2020).
45. Yao, C. *et al.* Benchmarking Reconstructive Spectrometer with Multiresonant Cavities. *ACS Photonics* 4c00915 (2024).
46. Xu, H., Qin, Y., Hu, G. & Tsang, H. K. Cavity-enhanced scalable integrated temporal random-speckle spectrometry. *Optica, OPTICA* **10**, 1177–1188 (2023).
47. Sun, C. *et al.* Scalable On-Chip Microdisk Resonator Spectrometer. *Laser & Photonics Reviews*, 2200792.
48. Yao, C. *et al.* Integrated reconstructive spectrometer with programmable photonic circuits. *Nat Commun* **14**, 6376 (2023).
49. Zhao, Y. *et al.* Miniaturized computational spectrometer based on two-photon absorption. *Optica,* **11**, 399–402 (2024).
50. Zhang, Z., Xiao, S., Song, Q. & Xu, K. Scalable on-chip diffractive speckle spectrometer with high spectral channel density. *Light Sci Appl* **14**, 130 (2025).
51. Zhang, Y., Xu, C., Zhao, Z., Su, Y. & Guo, X. Miniaturized chaos-assisted Spectrometer. *Light Sci Appl* **14**, 331 (2025).
52. Yao, C. *et al.* Chip-scale sensor for spectroscopic metrology. *Nat Commun* **15**, 10305 (2024).

53. Tsenkova, R., Atanassova, S., Ozaki, Y., Toyoda, K. & Itoh, K. Near-infrared spectroscopy for biomonitoring: influence of somatic cell count on cow's milk composition analysis. *International Dairy Journal* **11**, 779–783 (2001).
54. Bai, X. *et al.* Near-infrared spectroscopy and machine learning-based technique to predict quality-related parameters in instant tea. *Sci Rep* **12**, 3833 (2022).
55. Thanavanich, C., Phuangsaijai, N., Thiraphatchotiphum, C., Theanjumpol, P. & Kittiwachana, S. Instant quantification of sugars in milk tablets using near-infrared spectroscopy and chemometric tools. *Sci Rep* **12**, 18802 (2022).
56. Zaukuu, J.-L. Z. *et al.* Non-invasive prediction of maca powder adulteration using a pocket-sized spectrophotometer and machine learning techniques. *Sci Rep* **14**, 10426 (2024).
57. Natesan, S., Armenakis, C., Benari, G. & Lee, R. Use of UAV-Borne Spectrometer for Land Cover Classification. *Drones* **2**, 16 (2018).
58. Elmi, F., Movaghar, A. F., Elmi, M. M., Alinezhad, H. & Nikbakhsh, N. Application of FT-IR spectroscopy on breast cancer serum analysis. *Spectrochimica Acta Part A: Molecular and Biomolecular Spectroscopy* **187**, 87–91 (2017).
59. Yao, L., Tang, Y., Yin, Z., Pan, T. & Chen, J. Repetition rate priority combination method based on equidistant wavelengths screening with application to NIR analysis of serum albumin. *Chemometrics and Intelligent Laboratory Systems* **162**, 191–196 (2017).
60. Jessen, T. E. *et al.* Simultaneous determination of glucose, triglycerides, urea, cholesterol, albumin and total protein in human plasma by Fourier transform infrared spectroscopy: Direct clinical biochemistry without reagents. *Clinical Biochemistry* **47**, 1306–1312 (2014).
61. Henn, R. *et al.* Hemodialysis monitoring using mid-and near-infrared spectroscopy with partial least squares regression. *Journal of biophotonics* **11**, e201700365 (2018).
62. Jintao, X., Liming, Y., Yufei, L., Chunyan, L. & Han, C. Noninvasive and fast measurement of blood glucose in vivo by near infrared (NIR) spectroscopy. *Spectrochimica Acta Part A: Molecular and Biomolecular Spectroscopy* **179**, 250–254 (2017).
63. Heise, H. M., Delbeck, S. & Marbach, R. Noninvasive Monitoring of Glucose Using Near-Infrared Reflection Spectroscopy of Skin—Constraints and Effective Novel Strategy in Multivariate Calibration. *Biosensors* **11**, 64 (2021).
64. Yu, Y., Huang, J., Zhu, J. & Liang, S. An Accurate Noninvasive Blood Glucose Measurement System Using Portable Near-Infrared Spectrometer and Transfer Learning Framework. *IEEE Sensors Journal* **21**, 3506–3519 (2021).
65. Jain, P., Maddila, R. & Joshi, A. M. A precise non-invasive blood glucose measurement system using NIR spectroscopy and Huber's regression model. *Opt Quant Electron* **51**, 51 (2019).
