# Supplementary material for: Optical convolutional spectrometer
Source: Nat Photonics. 2026 Apr 15;20(6):664–72. doi: 10.1038/s41566-026-01891-6 (PMC13241319; doi:10.1038/s41566-026-01891-6)
Supplement: Supplementary file 2 — Reporting Summary [file 41566_2026_1891_MOESM2_ESM.pdf]

nature portfolio

Corresponding author(s): Qixiang Cheng
Last updated by author(s): Feb 18, 2026

Reporting Summary

Nature Portfolio wishes to improve the reproducibility of the work that we publish. This form provides structure for consistency and transparency in reporting. For further information on Nature Portfolio policies, see our Editorial Policies and the Editorial Policy Checklist.

Statistics

For all statistical analyses, confirm that the following items are present in the figure legend, table legend, main text, or Methods section.

n/a	Confirmed	
☐	☒	The exact sample size (n) for each experimental group/condition, given as a discrete number and unit of measurement
☐	☒	A statement on whether measurements were taken from distinct samples or whether the same sample was measured repeatedly
☐	☒	The statistical test(s) used AND whether they are one- or two-sided<br>*Only common tests should be described solely by name; describe more complex techniques in the Methods section.*
☐	☒	A description of all covariates tested
☒	☐	A description of any assumptions or corrections, such as tests of normality and adjustment for multiple comparisons
☐	☒	A full description of the statistical parameters including central tendency (e.g. means) or other basic estimates (e.g. regression coefficient) AND variation (e.g. standard deviation) or associated estimates of uncertainty (e.g. confidence intervals)
☒	☐	For null hypothesis testing, the test statistic (e.g. F, t, r) with confidence intervals, effect sizes, degrees of freedom and P value noted<br>*Give P values as exact values whenever suitable.*
☒	☐	For Bayesian analysis, information on the choice of priors and Markov chain Monte Carlo settings
☒	☐	For hierarchical and complex designs, identification of the appropriate level for tests and full reporting of outcomes
☒	☐	Estimates of effect sizes (e.g. Cohen's d, Pearson's r), indicating how they were calculated

Our web collection on statistics for biologists contains articles on many of the points above.

Software and code

Policy information about availability of computer code

Data collection	No software was used for data collection, as measurement data were directly obtained from the experimental device/setups.
Data analysis	The custom codes used for spectroscopic data analysis are publicly available at GitHub: https://github.com/GlitterinTechRnD/ConvSpec.<br>These codes were executed using Matlab (R2021b) and Python (v3.9).

For manuscripts utilizing custom algorithms or software that are central to the research but not yet described in published literature, software must be made available to editors and reviewers. We strongly encourage code deposition in a community repository (e.g. GitHub). See the Nature Portfolio guidelines for submitting code & software for further information.

Data

Policy information about availability of data

All manuscripts must include a data availability statement. This statement should provide the following information, where applicable:

- Accession codes, unique identifiers, or web links for publicly available datasets
- A description of any restrictions on data availability
- For clinical datasets or third party data, please ensure that the statement adheres to our policy

All data supporting this study are included within the main text and/or Supplementary Information. Source data are provided with this paper. They are also available in the University of Cambridge Repository at https://doi.org/10.17863/CAM.126013.

Research involving human participants, their data, or biological material

Policy information about studies with human participants or human data. See also policy information about sex, gender (identity/presentation), and sexual orientation and race, ethnicity and racism.

Reporting on sex and gender	Participants included both male and female adults, with the sex distribution reported in the Supplementary Information. No sex-based analysis was performed, nor considered an analytical factor in this work.
Reporting on race, ethnicity, or other socially relevant groupings	Race, ethnicity, and other socially defined groupings were not used as analytical variables in this study and were therefore not systematically collected or analyzed.
Population characteristics	For our spectroscopic measurements, the most relevant covariates lie in the inter-individual physiological differences that influence skin optical properties (such as hydration state, age, body fat rate, etc.). We acknowledge that such variability can influence the spectral signatures, and we explicitly point out in the manuscript that this inter-individual variability currently limits the transferability of our prediction model to unseen participants. However, given that the study focuses on demonstrating device performance and sensing capability rather than establishing population-level predictive models, we consider population-level covariate analysis to be beyond the scope of the present work.
Recruitment	Participants were recruited on a voluntary basis through local advertisement and word of mouth. To improve diversity in the dataset, we intentionally recruited male and female adult participants spanning a broad range of ages. We acknowledge that this recruitment approach introduces self-selection bias; however, the purpose of this study is to assess whether the spectrometer can reliably extract biomarker-related spectral information under real-world human conditions rather than to perform population-level statistical analysis. Such bias therefore does not affect the conclusions or the validation of device functionality.
Ethics oversight	Our study protocol was reviewed by the Institutional Ethics Committee of Xuzhou Medical University and deemed exempt from formal ethical review, given the non-invasive and minimal-risk nature of NIR spectroscopic sensing. Written informed consent from was obtained from all participants.

Note that full information on the approval of the study protocol must also be provided in the manuscript.

Field-specific reporting

Please select the one below that is the best fit for your research. If you are not sure, read the appropriate sections before making your selection.

☒ Life sciences ☐ Behavioural & social sciences ☐ Ecological, evolutionary & environmental sciences

For a reference copy of the document with all sections, see nature.com/documents/nr-reporting-summary-flat.pdf

Life sciences study design

All studies must disclose on these points even when the disclosure is negative.

Sample size	The study included measurements from n=126 participant sessions, yielding approximately 6,000 spectral datasets. In the context of near-infrared spectroscopy studies, this represents a comparatively large dataset. The sample size was chosen to ensure sufficient data for model training, evaluation, and device validation. The modelling results presented in the manuscript further support the rationale for this sample size.
Data exclusions	No data were excluded from the analyses.
Replication	In our biomarker sensing experiments, specific experimental workflows were designed for different biomarkers(as detailed in the Methods). Each participant completed an independent measurement process, generating paired spectral-biomarker datasets for subsequent modelling and device performance validation. Note that although the same workflow was replicated across participants, the aim of these experiments was not to reproduce specific biochemical outcomes, but to build up the scale of dataset.
Randomization	Randomization was not applicable to our study, as all participants underwent the same testing procedures.
Blinding	Blinding was not applicable to our study, as all measurements were conducted under standardized conditions and did not involve subjective assessments.

Reporting for specific materials, systems and methods

We require information from authors about some types of materials, experimental systems and methods used in many studies. Here, indicate whether each material, system or method listed is relevant to your study. If you are not sure if a list item applies to your research, read the appropriate section before selecting a response.

Materials & experimental systems

n/a	Involved in the study
☒	☐ Antibodies
☒	☐ Eukaryotic cell lines
☒	☐ Palaeontology and archaeology
☒	☐ Animals and other organisms
☒	☐ Clinical data
☒	☐ Dual use research of concern
☒	☐ Plants

Methods

n/a	Involved in the study
☒	☐ ChIP-seq
☒	☐ Flow cytometry
☒	☐ MRI-based neuroimaging

Plants

Seed stocks	No seed stocks were involved in our study.
Novel plant genotypes	Not applicable.
Authentication	Not applicable.
