# Supplementary material for: Optical convolutional spectrometer
Source: Nat Photonics. 2026 Apr 15;20(6):664–72. doi: 10.1038/s41566-026-01891-6 (PMC13241319; doi:10.1038/s41566-026-01891-6)
Supplement: Supplementary file 4 — Custom code used for different spectroscopic sensing applications, including the kNN and SVR models for solid classification and liquid concentration analysis, and the SVR and DFNN models for biomarker analysis. [file 41566_2026_1891_MOESM4_ESM.zip › kNN&SVR models for solid and liquid substances/Plastic/mangapls.pdf]

MANUAL OF THE PLS-GENETIC ALGORITHM TOOLBOX

by

RICCARDO LEARDI

(Dipartimento di Chimica e Tecnologia Farmaceutiche e Alimentari - University of Genoa)

(Additional comments by Mary Beth Seasholtz, Dow Chemicals)

5 main programs are contained in this toolbox:

- GAPLSOPT
- GAPLS
- GAPLSSP
- PLOTONE
- PLOTMORE

The programs have been written for Matlab 4 and then made compatible to higher releases. If possible, it is suggested to run them in Matlab 4, since they are much faster than in higher releases (about 40% if compared to Matlab 5).

GAPLSOPT has to be run in order to verify if GA can be used and to optimize the number of evaluations in each run, while GAPLS and GAPLSSP are the programs performing the variable selection (GAPLS for non spectral data, GAPLSSP for spectral data) and PLOTONE and PLOTMORE are the programs providing a graphical output.

It is strongly suggested to have a number of starting variables not greater than 200, in order to reduce the risk of overfitting. This can be achieved by “a priori” removing variables or, in case of spectra, by computing the average of n contiguous wavelenghts.

The data set must be a o,x+1 matrix, in which each line is an object, the columns 1:x are the X variables and the last column is the Y variable.

The default values are:
population size: 30 chromosomes;
on average 5 variables per chromosome in the original population;
5 deletion groups;
maximum number of variables selected in the same chromosome: 30;
probability of mutation: 1%;
probability of cross-over: 50%;
autoscaling;
maximum number of components: 15;
number of runs: 100;
backward elimination after 100 evaluations and at the end (if the number of evaluations is not a multiple of 100);
window size for smoothing (only in GAPLSSP): 3.

If the user wants to modify one of them, he has to modify the corresponding line in the GAPLS.M (or GAPLSSP.M) module.

The procedure usally followed is made by the following steps:

- Sort the objects in the matrix according to the Y variable. Since the cross validation is performed with 5 leave-out groups selected according to the 'venitian blind' method, this will make sure that the range and the dispersion of the Y is very similar for each deletion group (the importance of this step increases the lower the number of the objects, since in that case a random selection can produce quite unbalanced deletion groups).

- Gaplsopt(dataset,1). This does 50 runs with 100 evaluations with random permutations of the Y variable. The fitness function returned from these experiments is the average % variance explained in cross validation (if it is <0, then it is set to 0). This will tell whether we can proceed or not. If there are too few samples or too many variables the risk of overfitting is high, and in this case GA is not suggested. With good data sets values < 5 are obtained; as a rule of thumb, it can be said that GA can be safely applied when the result is < 10. Together with the numerical result, a plot is shown in which the progress of each run is plotted, together with the average value.

- Gaplsopt(dataset,2). This does 20 runs with 200 evaluations with "real" Y variables, followed by 20 runs each with a different random permutation. This estimates the number of evaluations that should be done in each run so as to get a good model without overfitting. In Figure 1 the behaviour of each run is shown as a function of the number of evaluations, together with the averages of the "true" and of the "randomized" runs and the difference between the averages. Figure 2 shows the difference as a function of the number of evaluations. The best way to decide the number of evaluations is to look at Figure 2 and to pick up the value from which no "significant" increase in the difference is observed; if it is lower than 50, select 50. The maximum is 200, since it has been noted that a higher number of evaluation does not give any substantial improvement.

- [b,c,d] = gapls(dataset, number of evaluations, precision). Use gapls for noncontinuous variables or gaplssp for continuous variables (e.g., spectra).
The input variable "precision" is optional; by knowing the prcision of the method more information will be given about the significance of the differences of the RMSECV.
b is the vector of the variables in decreasing order of selection
c is a matrix with the result of the final stepwise, in which:
    row 1 = number of variables used
    row 2 = response (% C.V. variance)
    row 3 = number of components
    row 4 = RMSECV
d is the vector with the frequency of selection of the variables
This program, having no interactive inputs, can be run several times as a batch procedure, having care of giving different names to the output matrices of each program (e.g., b1, b2, b3, …). Since the GA is a mainly stochastic algorithm, it is obvious that the final solutions of different GA's will not be exactly the same. By comparing the solutions from different GA's it is possible to have an idea of the robustness of the method. It is therefore suggested to run it at least 5 times for each data set.
For each program the user must decide on how many variables should be included in the model. Variables are added one at a time to the model in order of how many times they were selected during the 100 runs, and the cross validation results are obtained. Graphical information is provided by 4 plots. On all the plots a zooming can be obtained by clicking the left button of the mouse and dragging it. By clicking the right button the previous zooming will be obtained.
Figure 1 shows the histogram of frequency of selection of each variable (smoothed if using gaplssp). This will show which region(s) of the spectra are selected more often and which region(s) are virtually never selected. A green horizontal line shows the cutoff for the model with the minimum RMSECV (this will be referred to as "global model"). If a precision value is entered, a blue line shows the cutoff

for the model having the lowest number of variables among all the models for which the difference between their RMSECV and the minimum RMSECV is not higher than the precision (the "suggested model according to the precision criterion"). A green line shows the cutoff for the model having the lowest number of variables among all the models for which the difference between their RMSECV and the minimum RMSECV is not significant according to an F test ($p<0.1$) (the "suggested model according to the F criterion"). To avoid that a relevant decrease of RMSECV occurring immediately after the cutting levels is neglected, the following procedure has been implemented. Once the "suggested model" has been defined, the difference between its RMSECV and the RMSECV corresponding to the global minimum is computed. If the decrease of RMSECV occurring with the next complex model (usually having one more variable) is greater than 50% of the previously computed difference, then also this model is reported (as "better model") and gaphically shown with a dashed line. My personal advice is to select, among all the reported models (according to the two different criteria), the one with the highest number of variables.
Figure 2 shows the %CV explained variance as a function of the number of variables included. This should increase as variables are added, eventually reaching a maximum and holding relatively constant or decreasing. A green star indicates the global maximum, a red star indicates the value corresponding to the F-test and, if the precision value has been declared, a blue star indicates the value corresponding to the precision criterion.
Figure 3 shows the calibration spectra with lines corresponding to the regions selected by the global model (green), by the "precision" model (blue) and by the "F-test" model (red).
Figure 4 shows the RMSECV as a function of the number of variables included. Together with figure 2, this is the main graph for choosing the number of variables.
Printed to the screen is numerical information as well.

- In the case more programs have been run consecutively as a batch procedure, then only the plots regarding the last program can be displayed. To have the plots of the previous program, run Plotone(dataset,b,c,d,precision) on the b, c, d output vectors of each of them (precision is optional).

- After having selected the appropriate number of variables (and therefore the model) for each program, it is interesting to check how consistent the models are. After having created a new matrix with the b of all the programs (total = [b1;b2;b3;b4…];), run Plotmore(dataset,total). It will ask how many variables you want for each program. There will be a plot of the spectra, and lines showing which variables are selected for each program, top to bottom. We should have more here about how to decide on a final model, by selecting the spectral regions that are regularly selected.

- If the data set has been obtained by averaging contiguous wavelengths and if the window size used is considered to be too wide, then it is possible to remove the regions that have never been chosen; by doing that, a lower number of wavelengths will be retained and therefore it will be possible to use a smaller window size on the remaining data.

Summarizing it as a pseudocode, the standard procedure will be the following:

```
if more than 200 variables
  reduce the number of variables
end
gaplsopt(dataset,1);
if result > 10
  dataset not suitable for GA
end
```

```
gaplsopt(dataset,2);
define the number of evaluations (eval)
for i=1:numrep                              % 5 is the lowest suggested number of "replicates"
  if precision known
    [bi,ci,di]=gaplssp(dataset,eval,precision);    % for non-spectral variables run gapls
  else
    [bi,ci,di]=gaplssp(dataset,eval);               % for non-spectral variables run gapls
  end
end
for i=1:numrep
  plotone(dataset,bi,ci,di);
  select the number of variables for program i
end
tot=[b1;b2;…;bnumrep];
plotmore(dataset,tot);
if the window size applied for reducing the number of variables is acceptable
  select the spectral regions forming the final model
else
  remove the never selected regions and start again from the beginning
end
```

For further algorithmical and theoretical details please refer to:

R. Leardi and A. Lupiáñez, "Genetic algorithms applied to feature selection in PLS regression: how and when to use them", Chemolab, 41 (1998) 195-207.

R. Leardi, "Application of genetic algorithm-PLS for feature selection in spectral data sets", J. Chemometrics, 14 (2000) 643-655.
